# A review of defect engineering, ion implantation, and nanofabrication using the helium ion microscope

**DOI:** 10.3762/bjnano.12.52

**Published:** 2021-07-02

**Authors:** Frances I Allen

**Affiliations:** 1 Department of Materials Science and Engineering, University of California, Berkeley, CA 94720, USA; 2 California Institute for Quantitative Biosciences, University of California, Berkeley, CA 94720, USA

**Keywords:** defect engineering, focused helium ion beam-induced deposition, focused helium ion beam milling, helium ion beam lithography, helium ion implantation

## Abstract

The helium ion microscope has emerged as a multifaceted instrument enabling a broad range of applications beyond imaging in which the finely focused helium ion beam is used for a variety of defect engineering, ion implantation, and nanofabrication tasks. Operation of the ion source with neon has extended the reach of this technology even further. This paper reviews the materials modification research that has been enabled by the helium ion microscope since its commercialization in 2007, ranging from fundamental studies of beam–sample effects, to the prototyping of new devices with features in the sub-10 nm domain.

## Introduction

Since the helium ion microscope (HIM) was introduced 15 years ago [1–3], over one hundred HIMs have been installed worldwide and over one thousand research papers enabled by the HIM have been published. True to its classification as a microscope, and indeed its originally intended purpose, the HIM is widely used for microscopy. The microscopy functionality is primarily based on the detection of the secondary electrons that are generated by the finely focused ion beam as it is scanned across the sample. Compared with the scanning electron microscope (SEM), the HIM offers enhanced surface sensitivity, greater topographic contrast, and a larger depth of field [4–5]. A charge-neutralization system based on flooding the scanned region with low-energy electrons also permits high-quality imaging of electrically insulating materials, such as biological samples, thus avoiding the need for conductive coatings that can introduce artifacts and obscure nanoscale surface features [6–7]. Through extension of the technology to enable operation of the ion source with neon, sputtering at higher rates is made possible while retaining a small probe size. This has also opened the door to in situ materials analysis in the HIM using secondary ion mass spectrometry [8]. Further forms of materials analysis using the HIM include techniques based on the collection of backscattered helium ions and ionoluminescence [5].

Beyond microscopy and microanalysis, the HIM is also widely employed for materials processing, using the focused ion beam to intentionally modify the sample in some way. These operations essentially fall into two categories: At lower ion doses, various irradiation effects, such as defect formation and ion implantation, are used to locally change the properties of the material, and at higher doses, nanofabrication is performed using localized material removal (by sputtering) or addition (by gas-assisted deposition). Sometimes, lower-dose irradiation effects also lead to a nanofabrication outcome. For example, localized swelling by ion implantation can be used to pattern nanoscale surface topographies, ion-induced collisional mixing can restructure buried interfaces, and ion-induced chemical changes can be used for resist-based lithography. In the following, the field of materials modification research using the HIM is reviewed, subdivided into the following areas: 1. defect engineering, 2. ion implantation, 3. irradiation-induced restructuring, 4. resist-based lithography, 5. direct-write lithography/milling (including gas-assisted milling), and 6. gas-assisted deposition. Each topic is illustrated using a series of research highlights from the literature. In many cases, a particular application draws on the effects of more than one of the above areas, which is also discussed. At the root of all of these applications are the unique characteristics of the beam and its interaction with the sample. A quick recap of these details will be given first.

The intention of this review is to present the foundations and to summarize the state of the art. The list of referenced works is certainly not exhaustive in this extensive and rapidly evolving field. For previous reviews, the reader is referred to [9–13].

### Key features of the beam and the beam–sample interaction

**Figure 1:**
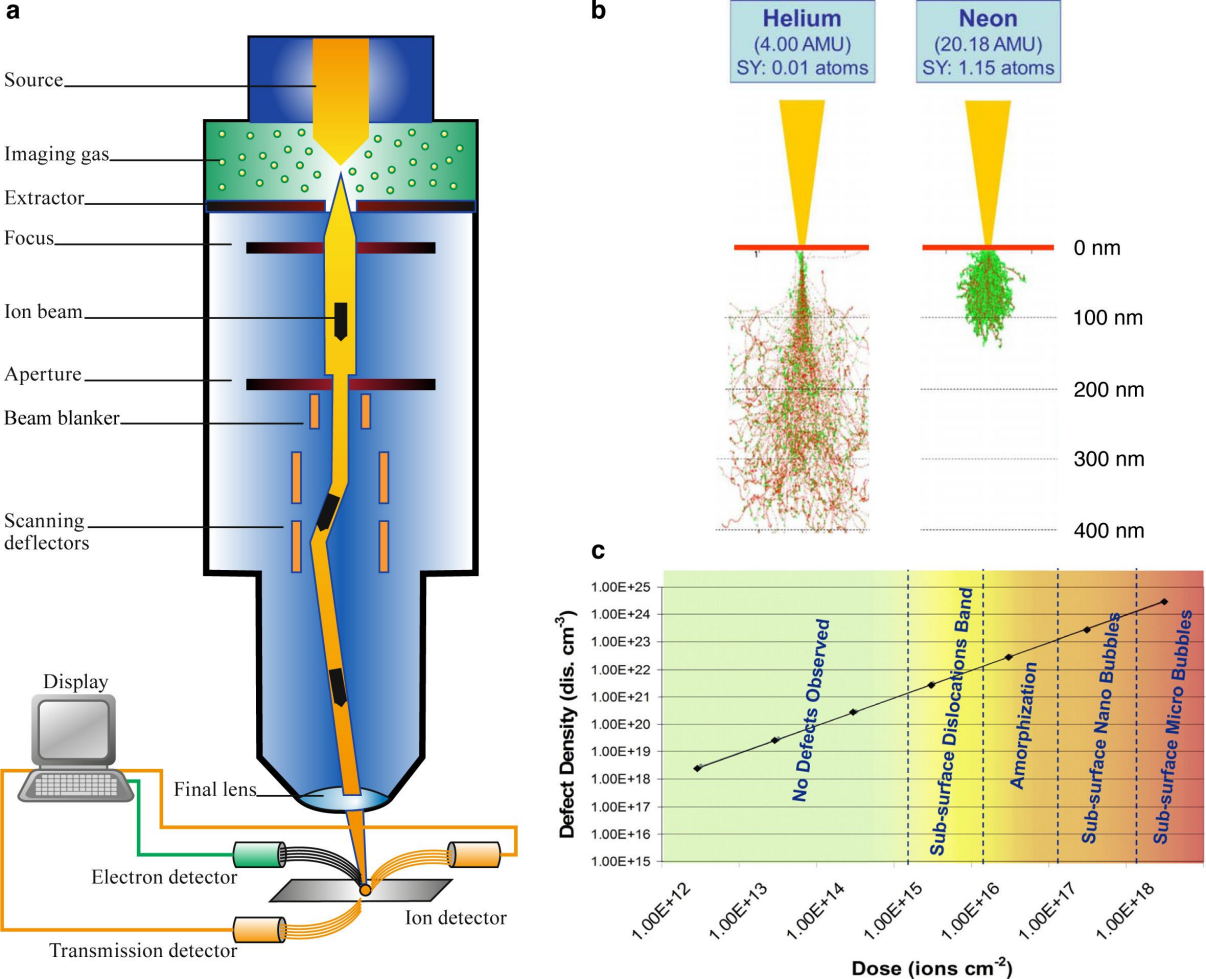
**(a)** Schematic diagram of the helium ion microscope. Adapted with permission from Cambridge University Press, [2], Morgan et al., An Introduction to the Helium Ion Microscope, Microscopy Today, 14(4), 24-31. Copyright 2006 Microscopy Society of America. **(b)** Simulated interaction volumes for 30 keV helium and neon ions incident on a silicon sample with sputter yield (SY) given for each case. Adapted with permission from [15]. Copyright 2010 American Vacuum Society. **(c)** Defect density versus dose for helium ions incident on bulk silicon and copper highlighting the threshold doses for various types of subsurface effects. Adapted with permission from [16]. Copyright 2009 American Vacuum Society. This content is not subject to CC BY 4.0.

The enabling foundation of all HIM applications, from imaging to nanofabrication, comes down to the unique characteristics of the beam and its interaction with the sample. The HIM ion beam is generated by a highly specialized gas field-ionization source (GFIS), comprising a tungsten needle with a three-sided pyramidal tip that is atomically sharp. The tip is held at high voltage and helium (or neon) gas is supplied to the source chamber. Ionization in the high-field region of the tip apex forms the positively charged ions that are extracted and delivered to the sample via the ion optical column (Figure 1a). Generally, the source is configured to run in the stable configuration of just three atoms at the tip apex (named the trimer), generating three beamlets. Via appropriate beam alignment and aperture selection, the emission from a single atom is selected. Since the source is atomically sharp, the virtual source size is exceptionally small and the source brightness is exceptionally high. With a small intrinsic energy spread of the helium ions of less than 1 eV, chromatic aberration effects are minimized, and with a de Broglie wavelength of just 0.08 pm, diffraction effects do not appreciably limit the ultimate beam size. Beam spot sizes on the sample down to 0.5 nm in diameter in the case of helium, and 2 nm for neon, are routinely obtained. For detailed discussions of the unique source characteristics of the HIM and its beam optics the reader is referred to [5,14].

In addition to the small probe size, the interaction volume of the helium ions (beam energy of typically 10–30 keV) is also characteristically narrow, especially over the first 100 nm or so in depth in the material (see Figure 1b). This means that defect creation, implantation, sputtering, and deposition can all be very localized. Correspondingly, the secondary electrons that are generated (escape depth of a few nanometers) emanate from an area not that much larger than the original impact area of the beam. These characteristics, together with a low yield of backscattered ions and therefore a very small amount of second-generation secondary electrons, enable high-resolution resist-based lithography and high-resolution ion beam-induced deposition with dramatically reduced proximity effects. In the case of neon (also shown in Figure 1b), there is more scattering close to the surface, but the advantage for milling is a sputter yield that is about two orders of magnitude higher than that of helium at the same beam energy [17].

Early HIM work by Livengood et al. investigated the interaction volume and resulting modification of the sample for helium ions impinging on bulk silicon and copper targets providing key insights into the beam–sample interaction [16]. Dose series were conducted and the interaction volumes directly visualized by preparing cross sections by gallium focused ion beam milling that were inspected by transmission electron microscopy (TEM). In this way, the microstructural effect of increasing the helium ion-induced defect density was probed, and threshold doses for a series of structural changes, such as amorphization and subsurface swelling, were established (Figure 1c). The motivation for this study at the time was to investigate the invasiveness of the helium ion beam with respect to nanofabrication tasks in the semiconductor industry. Yet, the impact of this work has been far-reaching, providing a valuable benchmark for a range of applications focused on either purposefully creating, or avoiding, varying degrees of disorder in a material. In the following, a range of applications based on these irradiation effects is described, starting with defect engineering studies at the lowest doses and then moving through higher-dose applications. The final applications using the highest doses (many orders of magnitude higher than the largest dose shown in Figure 1c) are milling and gas-assisted ion beam-induced deposition.

## Review

### 1 Defect engineering

The use of the HIM as a source of localized helium ion irradiation with which to tune material properties through the introduction of lattice defects has been demonstrated in a variety of nanoengineering applications. The generation of vacancies, preferential sputtering of one atomic species over another, and the introduction of increasing amounts of disorder leading to eventual amorphization of a crystalline material are all dials to turn. The properties engineered span electronic, magnetic, optical, chemical, and thermal properties. In the case of 2D and thin-film materials supported on a substrate, defects induced by backscattered ions and sputtered atoms also need to be considered. A recent computational analysis of these substrate effects can be found in [18].

#### Electrical and electronic properties

The majority of defect engineering studies using the HIM have focused on tuning electrical conductivity. First work in this area concentrated on graphene, seeking to locally modulate its 2D electronic structure through site-selective irradiation, rather than through chemical functionalization of its surface. Nakaharai et al. irradiated supported single-layer graphene with 30 keV helium ions increasing the dose from ca. 2 × 10^15^ to 1 × 10^16^ ions/cm^2^ (corresponding to an estimated vacancy defect concentration of 0.2–1.3%) and, within this dose range, measured a metal–insulator transition [19]. Raman analysis showed that even at the highest dose (i.e., for strongly insulating graphene), the crystal lattice structure of the graphene sheet was essentially preserved. A subsequent study by Moktadir et al. investigated the nature of the ion-induced defects further, and determined that after irradiation, the ion-induced vacancies become saturated with oxygen [20]. The authors of the latter thus proposed that the mechanism behind the irradiation-induced insulating behavior involved oxygen groups acting as charge traps that pin the Fermi level at the Dirac point.

Later conductivity tuning of graphene went on to combine this irradiation-induced effect with the fine patterning capabilities of the HIM performing line irradiations across graphene with varying step sizes between dwell points [21]. This produced continuously irradiated lines in the one extreme, and lines comprising a chain of separated points in the other. Conductivity analysis of these samples showed that in addition to the total dose, the scan strategy (which controls the uniformity of the dose and hence the uniformity of the resulting defects) can also be used to tune the local conductivity. A further study demonstrating the importance of factors other than the total dose investigated the effect of the size of the irradiated area [22]. Regions of monolayer graphene (again, supported) were irradiated using pattern shapes of different sizes, exploring lateral dimensions down to 10 nm, but all for the same total dose. It was found that the conductivities of the irradiated areas depended strongly on their geometrical size; for smaller areas, the same degree of insulating behavior as for the larger areas could not be achieved, even though the defect density was the same. The authors described a hopping carrier transport model to explain the effect and pointed out that the observed behavior essentially places a limit on the spatial resolution attainable when using the helium ion beam to selectively dose and thus change the conductivity of nanoscale regions. Several other studies of selective helium ion-induced conductivity changes in graphene have also been conducted [23] and a theoretical treatment of defect-induced conductivity changes upon helium ion irradiation of free-standing compared to supported monolayer graphene can be found in [24].

**Figure 2:**
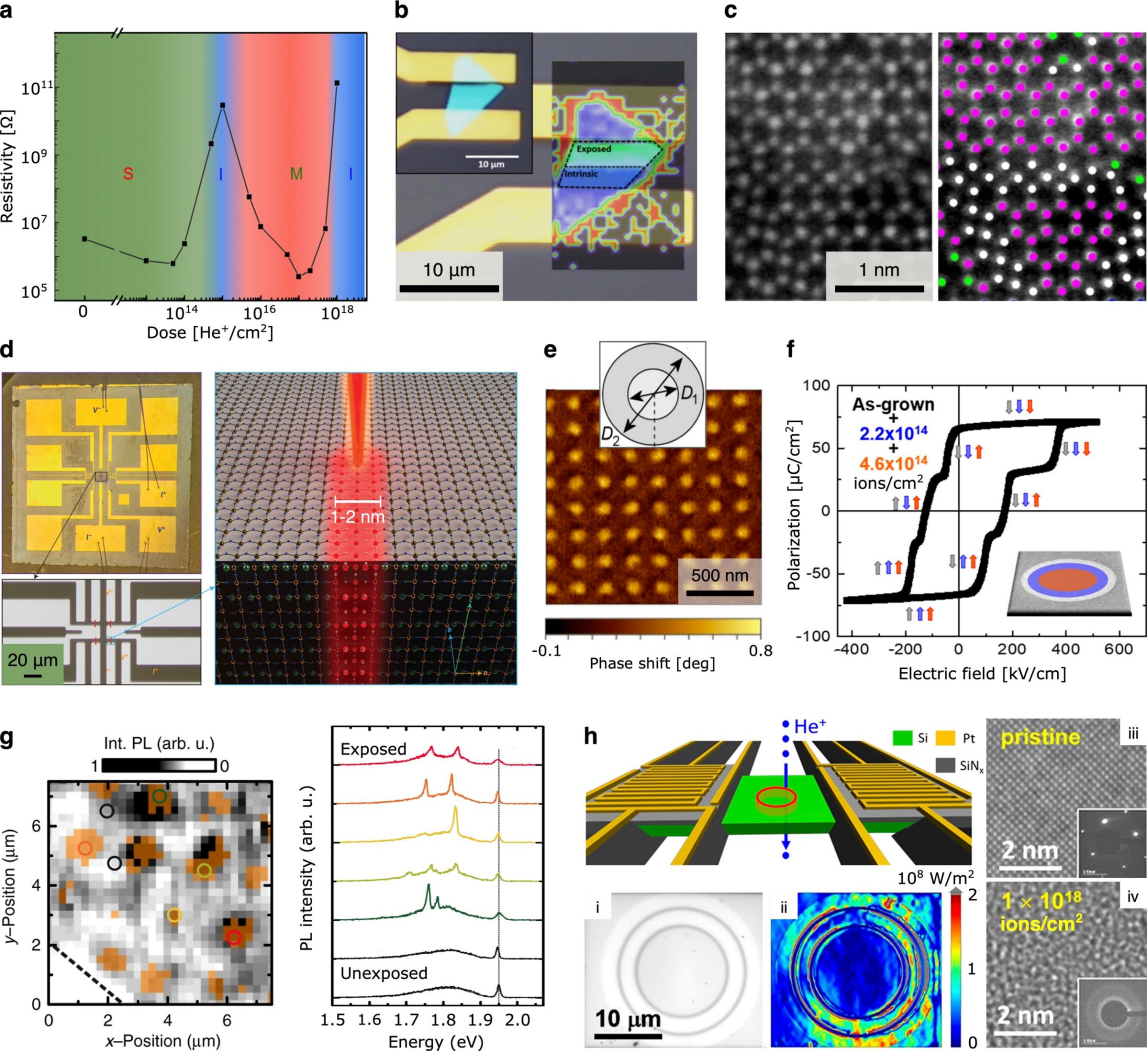
Defect engineering using the HIM. **(a)** Plot of resistivity versus dose for helium ion-irradiated MoS_2_ (few-layer) showing multiple phase transitions: semiconducting (S), insulating (I), metallic (M). Reprinted with permission from [25]. Copyright 2015 American Chemical Society. **(b)** Electronic homojunction in few-layer WSe_2_ created by site-selective helium ion irradiation: colorized Raman map on the right with optical micrograph of the device shown top left. Adapted from [29]. Copyright 2016 Stanford et al., licensed under a Creative Commons Attribution 4.0 International License, http://creativecommons.org/licenses/by/4.0/. **(c)** STEM-based analysis of atomic defects in single-layer MoSe_2_ created by helium ion irradiation. Adapted from [30]. Copyright 2016 Iberi et al., licensed under a Creative Commons Attribution 4.0 International License, http://creativecommons.org/licenses/by/4.0/. **(d)** Fabrication of Josephson junctions in a thin-film high-temperature superconductor by helium line irradiation: YBCO-based device with gold contacts (left) and schematic showing the irradiation approach (right). Adapted by permission from Springer Nature, [31], Nature Nanotechnology, Nano Josephson superconducting tunnel junctions in YBa_2_Cu_3_O_7−δ_ directly patterned with a focused helium ion beam, Cybart et al. Copyright 2015 Nature Publishing Group. **(e)** Magnetic force microscopy of a nanoscale array of discs with reduced magnetic anisotropy patterned into a Co/Pt multilayer: concentric helium ion irradiation scheme for each disc shown inset. Adapted from [32]. Copyright 2019 Sapozhnikov et al., licensed under a Creative Commons Attribution 4.0 International License, https://creativecommons.org/licenses/by/4.0/. **(f)** Multilevel ferroelectric switching of nanostructures fabricated by helium ion irradiation of a PbZr_0.2_Ti_0.8_O_3_ thin film using concentric patterns with variable dose. Adapted with permission from [33]. Copyright 2018 by the American Physical Society. **(g)** Site-selective formation of optically active defects in MoS_2_: photoluminescence (PL) map of an array created by localized helium ion irradiation (100 × 100 nm fields, pitch 2 μm) and corresponding PL spectra for regions marked with the colored circles. Adapted from [34]. Copyright 2019 Klein et al., licensed under a Creative Commons Attribution 4.0 International License, http://creativecommons.org/licenses/by/4.0/. **(h)** Demonstration of a microscale thermal cloak using helium ion irradiation to site-selectively tune the thermal conductivity of a suspended silicon membrane: schematic of device (top left), (i) optical image showing irradiated regions (darker contrast), (ii) corresponding heat flux map, (iii) TEM analysis of pristine silicon, (iv) TEM analysis of an irradiated region. Adapted with permission from [35]. Copyright 2019 American Chemical Society. The content of (a), (d), (f) and (h) is not subject to CC BY 4.0.

Various 2D transition metal dichalcogenides have also been the subject of conductivity-tuning studies in the HIM. For example, Fox et al. showed that site-selective helium ion irradiation, introducing point defects and local disorder, transformed targeted regions of a supported pristine few-layer MoS_2_ flake from semiconducting to insulating at a dose of ca. 1 × 10^15^ ions/cm^2^ [25]. The dose-versus-resistivity plot from this work is shown in Figure 2a. Upon increasing the dose to ca. 1 × 10^17^ ions/cm^2^, the material became amorphous, and the conductivity behavior changed to metallic. This was attributed to preferential sputtering of sulfur, increasing the relative amount of molybdenum in the material. Finally, at even higher doses, insulating behavior re-emerged, presumed to be due to excessive material removal by sputtering. In related work, a nearest-neighbor hopping mechanism mediated by the formation of extended metallic edge states in the defective lattice was proposed for the pseudometallic regime [26]. Line irradiation with helium ions bisecting a monolayer MoS_2_ flake has been shown to create a defective channel that can be used to fabricate a 2D memristive device [27]. And in a subsequent study, field-effect transistors based on monolayer MoS_2_ were irradiated with helium ions over the transistor channel and the effect of varying the size and position of the irradiated area on the electrical performance of the device was characterized [28].

Complimentary work on few-layer WSe_2_ by Stanford et al. also observed semiconductor–insulator–metal transitions for increasing dose, noting preferential sputtering of selenium [29]. Here it was found that for a given dose, hole transport was degraded more than electron transport. The authors went on to demonstrate a lateral p–n-like homojunction by irradiating just one side of a portion of WSe_2_ bridging two metal contacts to create semiconducting (pristine) and insulating (irradiated) halves contacted by the source and drain electrodes (Figure 2b). Direct-write of further logic circuits into single flakes of WSe_2_ and WS_2_ has also been demonstrated [26]. In addition, density functional theory has been used to model the effect of ion-induced defects on the electronic band structure of various 2D transition metal dichalcogenides [26,30,36], and band-excitation Kelvin probe microscopy has been used to probe the resulting changes in the local work function [30]. In several of these works, high-resolution scanning TEM (STEM) imaging has been performed to enable the analysis of the defects created on the atomic scale [26,29–30] (see, e.g., Figure 2c).

Apart from 2D materials, thin-film samples have also been the subject of electronic property tuning by localized helium ion irradiation. For example, using a helium ion dose of 5 × 10^14^ ions/cm^2^, permanent local tuning of the charge density in an amorphous thin film of the semiconductor indium gallium zinc oxide (film thickness 50 nm) has been demonstrated, thereby enabling activation of the electronic conduction properties of the material without needing to rely on thermal activation [37].

And finally in the category of electronic applications, a number of studies have used the HIM to direct-write Josephson tunnel junctions into thin films of high-transition-temperature superconductors. This was first demonstrated by Cybart et al. for the cuprate superconductor YBa_2_Cu_3_O_7−δ_ (YBCO), by scanning the helium ion beam in line mode across 4 μm wide strips of YBCO that had been locally pre-thinned to a thickness of 30 nm using argon ion milling [31]. Due to the high-resolution writing capability of the HIM, junction barriers of width 1–2 nm were created, that is, narrow enough for the tunneling current to propagate. An overview of the sample and a schematic outlining the irradiation approach are shown in Figure 2d. The electronic properties of the junction barrier can be continuously tuned by varying the irradiation dose, and the authors showed that by using irradiation doses of 2 × 10^16^ and 6 × 10^16^ ions/cm^2^, tunnel junctions with barriers exhibiting normal-metal and insulator behavior, respectively, were obtained. Similarly, the fabrication of superconducting quantum interference devices (SQUIDs) was demonstrated, using the helium line irradiation method to direct-write metallic and insulating Josephson junctions into pre-fabricated YBCO circuits [38].

By increasing the helium ion dose further, to the order of 10^17^ ions/cm^2^, highly resistive (amorphous) regions can be patterned into the YBCO thin film and thus be used to define precise sample geometries, without material removal. For example, high-dose irradiation over larger areas leaving a narrow gap in between has been used to define YBCO nanowires, which were then line-irradiated at lower dose to form nanowire-based Josephson junctions [39]. Similar multi-step irradiation routines have been used to fabricate SQUID micromagnetometers entirely using helium ion irradiation [40–43], nano-SQUID-based transimpedance amplifiers [44], and Josephson junctions in the superconducting bridge of spiral terahertz antenna structures [45]. The performance of Josephson junctions in YBCO thin films fabricated using the HIM has been further evaluated in [46].

Cross-sectional STEM analysis of helium line irradiations of YBCO has shown that the crystallinity of the Josephson junction barriers is essentially preserved, highlighting the high sensitivity of the electronic transport properties of YBCO to point defects introduced into the lattice [43]. The same study also confirmed that minimal lateral scatter of the ions in the YBCO thin film occurs; even at the higher doses used to induce amorphisity, the irradiated channels remain narrow and confined. In contrast, in a study based on another cuprate-based superconductor, La_1.84_Sr_0.16_CuO_4_ (LSCO), it was found that significantly higher doses (10^18^ ions/cm^2^) were necessary to induce resistive behavior [47], that is, an order of magnitude higher than the respective dose for YBCO. Moreover, significant lateral scatter of the helium ions in the LSCO thin film was observed, even for the lower doses, exacerbated by ion backscatter from the substrate interface. However, in the case of another high-temperature superconductor, MgB_2_, the creation of high-quality Josephson junctions by the HIM method has again been demonstrated [48].

#### Ferromagnetic properties

The first demonstration of defect engineering using the HIM was actually for a magnetic device by Franken and co-authors [49]. In this work, ferromagnetic microstrips comprising Pt(4 nm)/Co(0.6 nm)/Pt(2 nm) layers on an SiO_2_/Si substrate were irradiated on one half with 25 keV helium ions. It was found that at a dose of 2 × 10^15^ ions/cm^2^ a domain wall could be injected into the structure due to the introduction of lattice defects that locally reduced the perpendicular magnetic anisotropy. By raising the dose slightly to 3 × 10^15^ ions/cm^2^, the domain walls could then be pinned in a highly reproducible manner. The same publication compared these results with those obtained using gallium ion irradiation and found that domain wall pinning was more pronounced in the helium ion case. This was attributed to the higher resolution capability of the helium ion beam enabling the creation of a sharper energy barrier at the domain wall.

Later work in this area employed similar helium ion doses (1–4 × 10^15^ ions/cm^2^) to locally reduce the perpendicular magnetic anisotropy in a Co/Pt multilayered thin film [50]. Here, patterns comprising an arrangement of discs of 100 nm diameter with a pitch of 200 nm were irradiated, demonstrating the capability of the focused helium ion beam method to achieve magnetic property engineering in the form of a nanoscale periodic array. In follow-up work, consistent magnetization reversal for all disks was achieved by inhomogeneous irradiation using a concentric pattern with a higher dose on the outside, rather than using homogeneous irradiation [32] (Figure 2e). Nanoscale magnetic patterning of Co/Pt multilayers using a checkerboard helium ion irradiation pattern has also recently been demonstrated [51]. Furthermore, tuning of the metamagnetic transition temperature of an FeRh thin film (from antiferromagnetic to ferromagnetic behavior) has been achieved by varying the helium ion dose from 1 × 10^14^ to 5 × 10^15^ ions/cm^2^, with the patterning of features down to 25 nm in size [52].

In a study implementing doses up to 1 × 10^17^ ions/cm^2^, stripe patterns were irradiated onto a magnetic thin film structure (this time comprising a ferro- and an antiferromagnetic layer) [53]. The reason for using the higher doses was to investigate the effect of helium implantation and subsequent swelling of the layers and the underlying substrate on the magnetic modification obtained. It was found that helium ion bombardment influenced the magnetic anisotropy in both layers of the structure, strongly reducing the saturation magnetization of the layer system. Moreover, the behavior observed correlated with both the introduction of lattice vacancy defects, and with an increase in the average interatomic distances due to swelling, that is, a magnetovolume effect. And in a recent report implementing in situ current–voltage characterization, site-selective helium ion irradiation of cobalt-based magnetic multilayer structures has been used to modulate the local magnetic anisotropy, with multilevel switching achieved [54].

The neon ion beam of the HIM has also been used to tailor magnetic properties. The heavier ions were used to introduce chemical disorder into the target lattice through the local displacement of atoms in the collision cascade to create individual ferromagnetic domains in a previously non-ferromagnetic material [55]. In the cited work, Röder et al. used this phenomenon of disorder-induced ferromagnetism, together with the small probe size of the neon ion beam, to directly write nanomagnets into a chemically ordered non-ferromagnetic Fe_60_Al_40_ precursor film.

#### Ferroelectric properties

Similar to the magnetic anisotropy experiments described above, helium ion irradiation using the HIM has also been used to locally modify ferroelectric properties. For example, pinning of ferroelectric domains in PbZr_0.2_Ti_0.8_O_3_ thin films at doses of 0.22 × 10^15^ to 1 × 10^15^ ions/cm^2^ has been demonstrated, and through site-selective direct-write patterning with variable dose, various nanostructures with novel ferroelectric-switching functionalities have been fabricated [33] (Figure 2f). Using similar doses, out-of-plane piezoelectricity has been patterned into multilayered MoTe_2_ [56]. In the case of helium ion irradiation of a bulk van der Waals layered ferroelectric semiconductor crystal (CuInP_2_S_6_), local volume expansion due to helium ion implantation was observed, forming a conical surface topography within which for increasing dose the ferroelectric domains were gradually destroyed [57].

#### Optical properties

In early work concerned with tuning optical properties with the HIM, arrays of nitrogen-vacancy centers were fabricated in diamond [58–59]. Starting with a diamond sample that already contained substitutional nitrogen, the focused helium ion beam was used to create lattice vacancies at predefined locations (in a similar manner to the related electron beam-based methods). After annealing to allow for diffusion of the vacancies, the presence of nitrogen-vacancy centers was confirmed by photoluminescence. Conversely, helium ion irradiation of another material, silicon nitride, in the HIM was found to reduce its fluorescence [60]. This was attributed to ion beam-induced disruption of the optically active defects present in the material. In the cited work, the effect was used to locally reduce the fluorescence of patches of a silicon nitride membrane that were subsequently perforated by helium ion beam milling to create solid-state nanopores for biomolecule detection. Following this approach, a low fluorescence background was achieved, facilitating the translocation detection of fluorescently labeled biomolecules by optical means, as opposed to having to rely on the conventional ionic current method. Nanopore fabrication using the HIM is discussed in more detail in Section 5.

The HIM has also been used to tune the optical properties of quantum well structures [61]. In this work, epitaxially grown InGaAs/GaAs single quantum well structures were patterned with sets of stripes to emulate a grating structure, using doses of less than 10^12^ ions/cm^2^. The result was a periodic spatial modulation of the excitonic resonance of the quantum well perpendicular to the buried quantum well layer. Since the doses were so low, the change in optical properties was attributed to the local accumulation of defects (as opposed to collisional phase mixing). In a plasmonic application, resonant triangular nanostructures were created in a graphene sheet supported on SiO_2_/Si by selectively irradiating the graphene in the regions around the intended structures (note, the graphene was not milled away) [62]. Tuning of the resonant behavior of the nanostructures was demonstrated by adjusting the irradiation dose.

Closely related to the electronic property tuning of 2D transition metal dichalcogenides described earlier, irradiation-induced changes in the optical response of this class of materials has also been investigated using the HIM. This was first performed for few-layer WSe_2_ [29] and monolayer MoSe_2_ [30] (for the latter, changes in nanomechanical properties were also probed). In subsequent work on monolayer MoS_2_ by Klein et al., the effects of ion dose on the optical and valleytronic properties of the material were investigated [36]. In this study, Raman spectroscopy was used to systematically probe the effect of increasing disorder for increasing irradiation dose, and the corresponding distance between the ion-induced defects for each dose level was inferred. At lower doses, a redshift in disorder-related photoluminesce peaks was observed, attributed to chemisorption at monosulfur vacancies, whereas for doses higher than 10^14^ ions/cm^2^, a strong reduction in all photoluminescence peaks occurred, corresponding to high levels of disorder in the crystal. Up to this critical dose, it was shown that the valley polarization properties of the material were preserved (indicating that the electronic band structure of the semiconductor was largely unaffected). Only for high levels of disorder introduced into the system was the band structure degraded. Based on these results, the authors determined a critical dose for nanostructuring MoS_2_ below which the optical and valleytronic properties of the material are conserved. In subsequent work, the formation of arrays of optically active defects in monolayer MoS_2_ was demonstrated, using localized helium ion irradiation followed by encapsulation in hBN in order to enhance the optical quality of the defect states [34]. Results from this study are shown in Figure 2g. A recent study of the effect of helium ion-induced disorder on the Raman modes and photoluminscence behavior of bilayer MoS_2_ can be found in [63].

#### Chemical properties

Local helium ion irradiation has also been shown to modify the chemical properties of a material, for example, chemical etch rates. In HIM studies by Petrov et al. irradiating silicon nitride [64–65] and silicon dioxide [66–67] with 10^15^–10^16^ ions/cm^2^, the rate of subsequent wet-etching of the irradiated regions with hydrofluoric acid was found to increase by up to a factor of three (for Si_3_N_4_) and five (for SiO_2_). The change was attributed to ion-induced defects and demonstrates another potential form of HIM-enabled nanofabrication, namely site-specific ion-enhanced etching with high spatial resolution. Similarly, site-selective etching of MoS_2_ has been demonstrated using helium ion irradiation to create defective regions that become activated for oxygen adsorption and subsequent oxidative etching when heated in air [68].

Helium ion irradiation in the HIM, this time using a defocused beam to irradiate larger areas, has also been used to generate defects in exfoliated flakes of molybdenum dichalcogenides (MoS_2_ and MoSe_2_) to activate the catalytic activity of the basal planes of the crystal for hydrogen evolution reactions [69].

#### Thermal properties

Further defect engineering studies using the HIM have been concerned with tuning the thermal properties of materials. The first example of this demonstrated site-selective engineering of thermal conductivity along the length of individual nanowires [70]. Here, Zhao et al. irradiated discrete sections of free-standing crystalline silicon nanowires of 160 nm diameter to a series of target doses. It was found that a dramatic decrease in thermal conductivity occurred already at relatively low doses, corresponding to point defect concentrations of only a few percent. This result indicated a strong phonon scattering effect from the few point defects introduced. For higher doses above ca. 2 × 10^16^ ions/cm^2^, complete amorphization of the material occurred, although it was noted that the overall morphology of the structure was unaffected. This ability to locally tune the thermal conductivity by selective helium ion irradiation has since been used to manipulate heat flow in suspended crystalline silicon membranes, patterning concentric circles at variable dose to demonstrate a microscale thermal cloak [35] (Figure 2h).

In a subsequent study by Jin et al., dose-controlled localized helium ion irradiation has been used to introduce lattice defects into VO_2_ nanowires with which to control the conduction mechanisms of thermal and electrical transport in this material [71]. By performing a dose series and measuring the thermal and electrical conductivities of helium ion-irradiated VO_2_ around its insulator–metal transition temperature, a fundamental study of the contradictorily low electronic thermal conductivity of VO_2_ in the metallic regime was enabled. Similarly, Bi_2_Te_3_ nanoribbons have been irradiated with helium ions in order to defect-tune conduction mechanisms and study the anomalously high electronic thermal conductivity of this material [72].

#### Additional defect engineering studies

Defect formation in supported and free-standing multiwalled carbon nanotubes upon irradiation with both 25 keV helium and neon ions has also been investigated, since ion (and electron) irradiation can be used to modify the mechanical, electronic, and magnetic properties of these materials [73]. In this work, the focus was on characterizing the accumulation of defects and structural changes for increasing dose, using correlative Raman spectroscopy and TEM. The effect of sample thinning due to sputtering on the overall damage evolution process was also noted.

### 2 Ion implantation

Helium ion implantation and the associated structural changes to a material are often unwanted, with measures taken to avoid these effects. However, the HIM also offers a unique opportunity for fundamental studies of helium ion implantation phenomena (such as the formation of helium nanobubbles and blisters), as well as the opportunity to leverage these phenomena for practical applications. Using the HIM, individual grains/interfaces/regions of interest can be irradiated with a known dose of ions for systematic studies on the nanoscale. This brings certain advantages compared with the traditionally used large-scale experiments. For example, although experiments using ion accelerators and plasma ion sources enable investigations over a wide range of beam energies and give the experimenter much freedom in terms of sample environment, the beam spot sizes are large (typically hundreds of micrometers), preventing study of the irradiation response of individual micro- and nanoscale features. In addition, the large-scale experiments are much less controlled in terms of dose, whereas with the HIM, systematic repeatable studies over many samples can be performed. In fact, at doses below the threshold for nanobubble formation, the introduction of helium atoms into interstitial sites can be used to delicately induce strain into a crystal lattice, which can be leveraged for strain engineering. And at higher doses, localized and controlled surface swelling can be used to create 3D structures from an initially flat substrate, that is, ion implantation using the HIM can also be used for nanofabrication.

#### Strain engineering

When helium ions are implanted into a crystal lattice they insert as atoms into interstitial sites, and due to their chemical inertness, do not react with the material. This interstitial placement induces strain into the lattice and hence offers a method for so-called strain engineering of a range of physical properties. Very recently, ion implantation using the HIM has been investigated for this purpose, demonstrating the patterning of out-of-plane strain into epitaxial thin films of bismuth ferrite [74]. In this work, Toulouse et al. showed that by continuously varying the dose, continuous elongation of the unit cell in the out-of-plane direction could be achieved, thus enabling fine control over the strain induced. Future work in this direction using the high-resolution patterning capabilities of the HIM for highly tunable strain engineering on the nanoscale is to be expected. Related to this are the ion implantation-induced membrane folding studies discussed at the end of Section 3.

#### Helium nanobubbles and blisters

**Figure 3:**
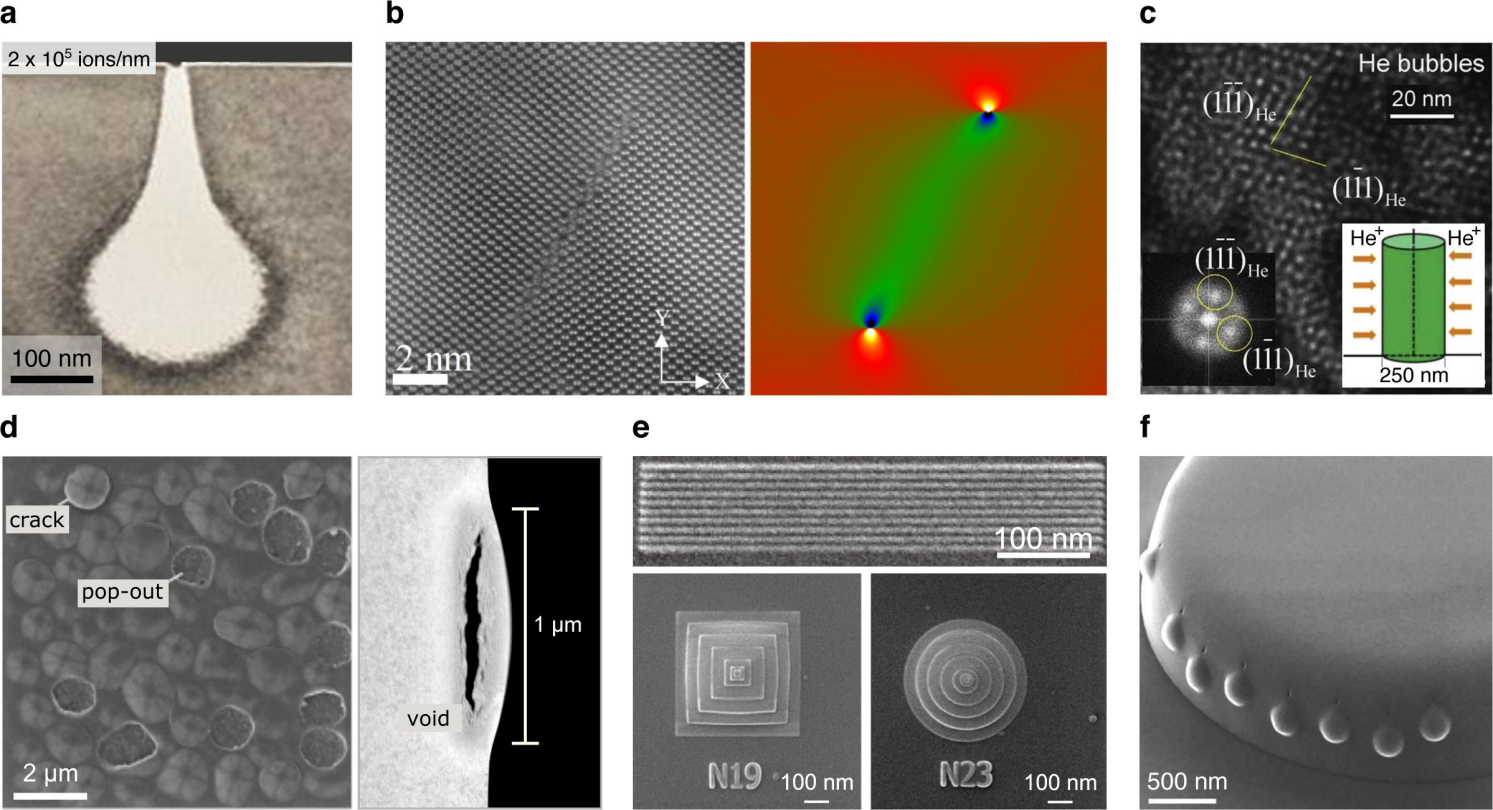
Effects of helium ion implantation studied with the HIM. **(a)** TEM cross-section analysis of the implant profile of 35 keV helium ions (line scan) in silicon for a dose above the amorphization threshold (the bright teardrop-shaped volume is the amorphized region). Adapted with permission from [75]. Copyright 2014 American Vacuum Society. **(b)** STEM image of a silicon crystal irradiated with helium ions in line-scan mode (left) with corresponding strain field analysis (right). Adapted with permission from [76]. Copyright 2019 American Vacuum Society. **(c)** Helium gas-bubble superlattice formed in copper: TEM image showing helium nanobubbles (bright contrast) with corresponding fast Fourier transform; schematic outlining the implantation principle from opposite sides of a flipped nanopillar shown inset. Adapted from [78]. Copyright © 2016 Acta Materialia Inc. Published by Elsevier Ltd. [78] is an open access article under a Creative Commons Attribution 4.0 International License (http://creativecommons.org/licenses/by/4.0/). **(d)** HIM image of helium blisters formed on tungsten (left) and STEM cross-section analysis of a single blister (right). Adapted from [84], Scripta Materialia, 178, Allen et al., Key mechanistic features of swelling and blistering of helium-ion-irradiated tungsten, 256-260, Copyright (2019) Acta Materialia Inc., with permission from Elsevier. **(e)** Helium nanoscale tumefaction to create raised lines and pyramid/cone structures in silicon. Adapted from [94], Zhang et al., Direct-write three-dimensional nanofabrication of nanopyramids and nanocones on Si by nanotumefaction using a helium ion microscope, Nanotechnology, Vol. 26(25), 255303, first published 4 June 2015. Copyright © 2015 IOP Publishing Ltd. Reproduced with permission. All rights reserved. **(f)** Helium point irradiations into the side of a diamond microscale disk creating precise surface protrusions. Adapted from [95], Kim et al., Focused-helium-ion-beam blow forming of nanostructures: radiation damage and nanofabrication, Nanotechnology, Vol. 31(4), 045302, first published 24 October 2019. Copyright © 2019 IOP Publishing Ltd. Reproduced with permission. All rights reserved. The content of (a), (b), (d), (e) and (f) is not subject to CC BY 4.0. For (c), please see the copyright notice in the caption.

At higher doses, the vacancies and interstitials that form in a crystalline material upon helium ion irradiation can diffuse and combine to form helium nanobubbles. And upon increasing the irradiation dose further, nanobubbles can coalesce to form larger cavities. Eventual rupture of cavities formed in near-surface regions can then lead to surface blistering. These phenomenona have been known and investigated for several decades. In early HIM studies on the subsurface effects of helium ions on bulk silicon and copper targets by Livengood et al., the formation of nanobubbles and larger subsurface voids was indeed also observed [16]. Threshold doses for the formation of a subsurface dislocation band, for the onset of amorphization, and for the formation of nanobubbles and larger voids, were established (as shown earlier in Figure 1c). Tan et al. performed detailed TEM cross-sectional analysis of the implantation profiles from line exposures [75] (Figure 3a). A recent extension of this work has been reported by Li et al. [76], which includes strain field analysis (Figure 3b) and a survey of bubble size distributions. In work using a gold target, Veligura et al. investigated the effect of varying the beam energy on the subsurface damage process [77]. For high-dose implantation at high beam energies (35 keV), blister formation was observed, as in [16]. This is explained by the formation and accumulation of helium nanobubbles beneath the surface that cannot escape, and which coalesce to form a subsurface cavity (blister). However, at lower beam energies (15 keV), the authors observed that the helium nanobubbles formed closer to the surface, creating a porous structure via which helium can then escape and blister formation is avoided [77].

A further interesting effect of helium ion irradiation of crystalline materials under certain conditions is the formation of a so-called gas bubble superlattice, comprising a periodic 3D array of nanobubbles imposed onto the host lattice. This can result in so-called radiation hardening of the material, which is of particular significance in the development of radiation-tolerant structural materials for deployment in nuclear fission and future nuclear fusion reactors. It has been shown that the gas bubble superlattice can be created by both broad beam or plasma exposure, as well as by repeated scanning of a selected area with the focused helium ion beam of the HIM. Wang et al. used the HIM to conduct a systematic study of the effect of such a helium gas bubble superlattice on the mechanical properties of copper, which is representative of a range of fcc alloys that are known to exhibit radiation tolerance [78]. Copper nanopillar specimens were irradiated at room temperature with known doses of helium ions to generate the gas bubble superlattice (Figure 3c) and subsequently nanomechanical compression testing of the implanted structures was performed by in situ TEM to enable direct and quantitative observation of the superlattice during deformation.

Other materials of interest for nuclear reactor design that have been the subject of HIM irradiation studies include silicon carbide grains in a pyrolytic carbon matrix [79], α-LiAlO_2_ pellets [80], tristructural-isotropic fuel particles [81], oxide dispersion-strengthened steels [82], tungsten [83–85], an Fe–Zr alloy [86], a Y_2_O_3_/Fe bilayer [87], and nanocluster films of magnetite and core–shell iron–magnetite nanoparticles [88]. In these studies, various implantation effects have been investigated, including the tendency for grain boundaries and interfaces to act as sinks for irradiation-induced defects and implanted ions [79–80,82–83,86–87], and at higher doses, the subsurface swelling that results in blistering and delamination [84] (Figure 3d), which for deployment of a material in a reactor needs to be avoided [80,82–85].

Returning to silicon, the nanomechanical properties of helium ion-irradiated silicon nanopillars have since been investigated as well, observing a softening behavior upon amorphization and swelling [89]. Helium ion irradiation of single-crystal diamond nanopillars has revealed an orientation dependence of the irradiation damage and associated mechanical response, with the typical swelling also observed [90]. In contrast, studies of both helium and neon ion-irradiated polymers have shown that swelling does not occur at the threshold doses observed for crystalline targets, attributed to the significantly higher diffusion coefficients of the noble gas atoms in the polymer, allowing the gas to diffuse out rather than accumulate [91]. In fact, a study has used localized helium ion irradiation to locally shrink a polymer and thereby modify the height of metallic thin films supported on a polymer substrate [92]. This effect was attributed to ion-induced scission of the polymer chains and subsequent cross-linking and compaction, which can be understood in terms of the dominance of energy losses by electronic scattering in the case of light ions such as helium, compared with the nuclear scattering that dominates energy loss for heavier ions. Yet, even in polymeric materials, swelling under implantation with high doses can still occur, and in general, the above studies underscore the advantage for many nanofabrication applications of a sample that is thinner than the ion stopping distance such that implantation effects can be avoided [93]. Although in some cases subsurface swelling is specifically desired, as described further below.

#### Nanofabrication using subsurface swelling

The localized volume expansion observed for a range of crystalline materials upon helium ion irradiation at high dose has also been put to use for several nanofabrication tasks. For example, irradiation of silicon substrates has been used to pattern raised lines, achieving a half-pitch down to 3.5 nm [94] (Figure 3e, top). In the same study, 3D nanopyramids and nanocones rising from the silicon surface were also created, achieved by dosing a series of concentric square frame and annulus patterns, respectively, with increasing dose from the perimeter to the center (Figure 3e, bottom). This tumefaction technique has the potential to be used to fabricate molds for nanoimprint lithography. In a recent study, localized helium ion implantation into diamond nanostructures using point or line-scan exposures has been employed for nanoscale “blow-forming”, producing precise surface protrusions in the regions targeted [95] (Figure 3f).

Finally, helium ion-induced swelling of a substrate can also be used to intentionally deform a supported structure. For example, a silicon substrate supporting an amorphous nanoporous aluminum oxide structure was irradiated with helium ions causing dome-shaped swelling of the substrate and thereby 3D deformation of the supported material, accompanied by enlargement of the nanopores to accommodate the new topography [96].

### 3 Irradiation-induced restructuring

The following applications have used localized helium ion irradiation in the HIM to change the morphology and/or internal structure of a material by ion-induced mass transport along surfaces, ion-induced collisional atomic mixing, and through stress gradients induced in membrane targets by ion implantation.

#### Ion-induced mass transport

**Figure 4:**
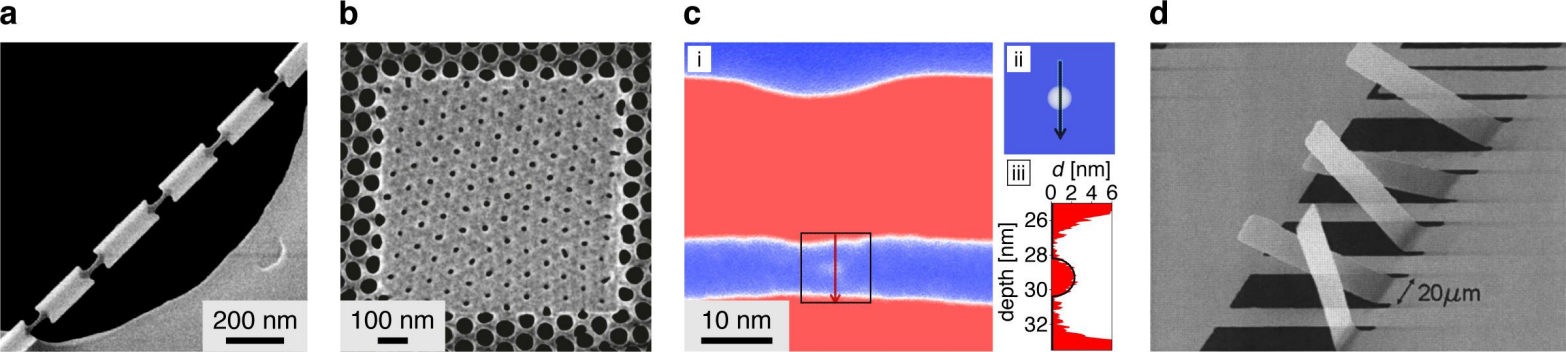
Examples of restructuring using localized helium and neon ion irradiation. **(a)** Localized thinning of a GaAs nanowire by site-selective helium ion irradiation at low dose: The proposed mechanism is mass transport by surface diffusion. Adapted from [97]. Copyright © 2017 WILEY-VCH Verlag GmbH & Co. KGaA, Weinheim. Used with permission from Aramesh, M., Ion-Beam Sculpting of Nanowires, physica status solidi (RRL)-Rapid Research Letters, John Wiley and Sons. **(b)** Related pore shrinking of nanoporous amorphous aluminum oxide by helium ion irradiation. Adapted from [96]. Copyright 2018 Aramesh et al., licensed under a Creative Commons Attribution 4.0 International License, http://creativecommons.org/licenses/by/4.0/. **(c)** Collisional mixing creating a silicon nanoparticle in a buried SiO_2_ layer by localized neon ion irradiation: (i) energy-filtered TEM map, (ii) simulated projection thickness of the silicon nanoparticle, (iii) measured projection thickness (red) and simulated (black). Adapted from [98]. Copyright 2018 Xu et al., licensed under a Creative Commons Attribution 4.0 International License, http://creativecommons.org/licenses/by/4.0/. **(d)** Membrane folding by localized helium ion irradiation using different beam energies at the base of free-standing silicon nitride cantilevers. Reprinted with permission from [99]. Copyright 2007 American Vacuum Society. The content of (a) and (d) is not subject to CC BY 4.0.

In one example of a morphological change attributed to ion-induced mass transport, segments of a free-standing GaAs nanowire of 100 nm diameter were irradiated locally with 30 keV helium ions at relatively low dose resulting in local thinning of the wire [97] (Figure 4a). Given the low dose applied, sputtering alone could not account for the dimension reduction observed. In fact, thickening of the adjacent unexposed regions was noted, thus adding weight to the hypothesis that ion-induced mass transport by surface diffusion was primarily responsible for the thinning effect encountered. In a related study, helium ion irradiation of an amorphous nanoporous aluminum oxide sample caused pores to markedly shrink in size, also attributed to ion-induced mass transport [96] (Figure 4b).

Further related to mass transport, local helium ion irradiation has been used to induce the growth of semiconductor nanowires [97]. In this study by Aramesh, gold catalyst nanoparticles were distributed onto GaAs and InAs substrates and upon irradiating selected regions with helium ions, semiconductor nanowires grew from the gold nucleation sites. By increasing the beam energy and current, the growth of single-crystal nanowires was also shown. This is reminiscent of nanowire growth by the vapor–solid–liquid mechanism, except here the process was performed at room temperature and without the flow of a gaseous precursor. In the HIM case it was proposed that the nanowire growth process was catalyzed by a combination of irradiation-induced diffusion and local ionization.

#### Ion-induced collisional mixing

Another form of restructuring, this time using the neon ion beam, was demonstrated by Xu et al. using a collisional ion beam-induced mixing technique [98]. Here, neon line irradiation of an Si(25 nm)/SiO_2_(6.5 nm)/Si(bulk) stack was used to induce collisional mixing of silicon atoms into the buried SiO_2_ layer. Upon subsequent thermal annealing, 1D chains of silicon nanocrystals of 2.2 nm diameter self-assembled in the center of the SiO_2_ layer. A TEM-based analysis of an isolated silicon nanocrystal from this work is shown in Figure 4c. The authors highlighted that the demonstration presents a promising new technique for the fabrication of single-electron transistor devices. This work is also closely related to the magnetic property tuning by Röder et al. described in Section 1, where localized neon ion irradiation was used to create ferromagnetic domains by introducing chemical disorder as a result of the ion-induced nuclear collision cascade [55].

In a further study, ion beam-induced mixing using both helium and neon ions in an Mo/Si multilayer beneath a 50 nm nickel layer was reported [100]. This was in fact a milling study for mask repair for extreme-ultraviolet (EUV) lithography applications (nickel being a candidate EUV mask absorber material, and Mo/Si being the EUV mirror). In this particular case, the observed mixing effect of atoms between the layers was undesired.

#### Membrane folding

Recently, several papers on FIB-enabled nanoscale kirigami have been published using the gallium FIB to mill line patterns in free-standing membranes (equivalent to traditional macroscale paper cuts) followed by localized gallium ion implantation to induce stress gradients across the membrane that lead to deformation by buckling [101]. Various intricate 3D structures have been created following this approach. Interestingly, right at the beginning of HIM-based research, membrane folding using localized helium ion irradiation was also demonstrated [99] (Figure 4d). In this work by Arora et al., free-standing cantilevers in silicon nitride membranes that had been prepared by photolithography were irradiated near the base with helium ions at different energies. Upwards versus downwards bending was observed, depending on the implantation depth profiles. It is conceivable that by using the HIM to both mill the cuts and induce the buckling, the size of FIB-based nanoscale kirigami structures can be further reduced compared to what is currently possible using the gallium FIB technique. First steps in this direction have recently been taken [102].

### 4 Resist-based lithography

The patterning of mask structures for resist-based lithography also relies on irradiation effects. Here, the goal is to create a 2D or 3D structure through a templating approach, whereby a chemical change is induced in the resist layer causing it to become either insoluble (negative tone resist) or soluble (positive tone resist) in the subsequent development step. The drive to fabricate devices with ever reducing dimensions has meant that alternatives to diffraction-limited photon-based lithography have evolved, using charged particle beams [103]. Electron beam lithography (EBL) can achieve minimum features sizes approaching 5 nm, but key drawbacks of using electrons (beam energies typically up to 100 keV) are the low sensitivity of the resists to these particles and also proximity effects (i.e., unintentional exposure of resist surrounding the targeted pixel) due to backscattered electrons from the substrate and the secondary electrons they generate. In contrast, for ion beam lithography, the sensitivity gain can be orders of magnitude higher and the proximity effect is dramatically reduced, due to the much lower propensity of the ions to backscatter. However, before the introduction of the HIM, ion beam-based lithography mainly relied on the gallium FIB, for which major drawbacks were ion beam sputtering of the resist and the relatively large beam spot size (several nanometers) with its significant beam tails. Helium ion beam lithography (HIBL) using the HIM is therefore a lucrative alternative, combining the benefits of the tightly focused probe and low sputter yield of the light ions with the high resist sensitivity and greatly reduced proximity effect afforded by the ion beam-based method. Reviews of HIBL can be found in [104–105].

#### Sub-10 nm patterning

**Figure 5:**
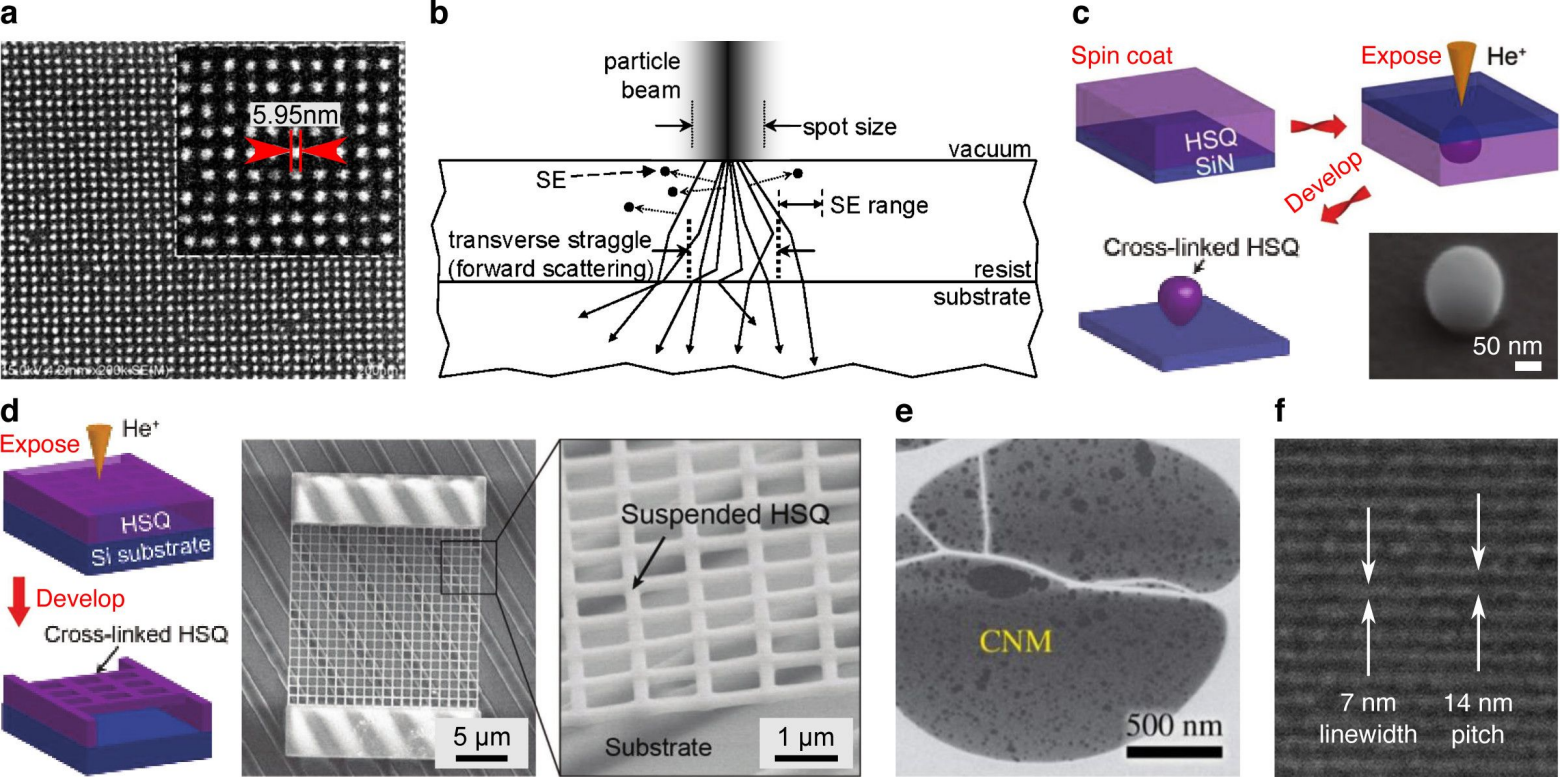
Resist-based lithography using helium and neon ions. **(a)** 6 nm dots on a half-pitch of 7 nm fabricated in HSQ by HIBL. Adapted with permission from [106]. Copyright 2009 American Vacuum Society. **(b)** Schematic summarizing the beam–sample interactions involved in resist exposure (ion backscatter from the resist–substrate interface is negligible). Reprinted with permission from [107]. Copyright 2009 American Vacuum Society. **(c)** Procedure for the 3D measurement of the point-spread function for HIBL using a through-membrane exposure technique: The SEM image shows a cross-linked volume of HSQ upon development of the resist following the point exposure. Adapted from [108]. Copyright © 2018 WILEY-VCH Verlag GmbH & Co. KGaA, Weinheim. Used with permission from Cai et al., 3D Volumetric Energy Deposition of Focused Helium Ion Beam Lithography: Visualization, Modeling, and Applications in Nanofabrication, Advanced Materials Interfaces, John Wiley and Sons. **(d)** Fabrication of a suspended mesh structure by direct exposure of the resist from the front side. Adapted from [108]. Copyright © 2018 WILEY-VCH Verlag GmbH & Co. KGaA, Weinheim. Used with permission from Cai et al., 3D Volumetric Energy Deposition of Focused Helium Ion Beam Lithography: Visualization, Modeling, and Applications in Nanofabrication, Advanced Materials Interfaces, John Wiley and Sons. **(e)** Carbon nanomembrane (CNM) fabricated by HIBL of aromatic self-assembled monolayers. Reprinted from [112]. Copyright 2014 Zhang et al., licensed under a Creative Commons Attribution 4.0 International License, http://creativecommons.org/licenses/by/4.0/. **(f)** 7 nm lines on a half-pitch of 7 nm fabricated in HSQ by NIBL. Adapted with permission from [113]. Copyright 2011 American Chemical Society. The content of (a), (b), (c), (d) and (f) is not subject to CC BY 4.0.

Resist-based HIBL was already investigated in the early years of the HIM and the fabrication of sub-10 nm structures was soon demonstrated [106–107]. Moreover, remarkably dense patterning of sub-10 nm features was achieved. For example, Sidorkin et al. demonstrated patterning of 6 nm dots with a half-pitch of 7 nm in HSQ resist [106] (Figure 5a). These results showcased both (1) the high patterning resolution of individual features, made possible by the subnanometer probe, minimal lateral scatter of ions in the resist, and associated narrow spatial distribution of secondary electrons, as well as (2) the greatly reduced proximity effect, due to negligible backscatter of the helium ions and thus negligible presence of second-generation secondary electrons, that together would otherwise cause feature blurring. The beam–sample interactions involved in the exposure of the resist are summarized schematically by Winston et al. in Figure 5b [107]. Modeling and experimental measurements of the 2D point-spread function for HIBL (i.e., the spatial distribution of energy deposition, which determines the proximity effect) can be found in the same reference. Cai et al. extended this work to a 3D visualization of the point-spread function by performing point exposures on HSQ through a thin layer of silicon nitride and then developing the resist to remove non-exposed regions, leaving drop-shaped cross-linked structures that define the 3D exposed volumes [108] (Figure 5c). The authors also directly exposed substrate-supported resists from the front side and fabricated hollow and suspended nanostructures (see Figure 5d) by appropriate choice of beam energy and dose during the patterning process. A further study has investigated the HIBL proximity effect for large-area exposures, as well as the potential to use HIBL to pattern tilted samples, making use of the large depth of field of the HIM [109]. A model-based method for HIBL proximity-effect correction has also been developed [110]. And a recent study has investigated the resist sensitivities and ion scattering profiles for 30 keV helium ions compared with a few heavier ions of similar energies [111].

A number of measurements have been made of the dose required to achieve HIBL full exposure of EBL-standard HSQ and PMMA resists, consistently finding that compared with electrons, one to two orders of magnitude lower doses of helium ions are required [114–116]. The high sensitivity to helium ions of the inorganic HafSOx resist (designed for EUV lithography) has been explored [117] and the unique potential to use HIBL to pre-screen EUV resists has been evaluated [118–120]. The advantages of HIBL have also been highlighted using a number of other novel resists, including a fullerene-based molecular resist [121], a tetracene molecular resist [122], and various organic–inorganic resists [123–129]. The enhanced sensitivity of resists to helium ions has been attributed to the higher secondary electron emission yield and the fact that the helium ions deposit their energy over much shorter distances in the resist, thus increasing the local energy density.

HIBL has also been combined with nanoimprint lithography, achieving lines down to a half-pitch of 4 nm [130]. In this technique, the helium ion beam is first used to pattern a resist to make a master template. The template is then pressed into a second resist, which is cured by UV exposure before the master template is retracted and re-used. Hereby, the high resolution and the low proximity effect of HIBL are combined with the high-throughput and low-cost benefits of nanoimprint. In another HIBL-related study, helium ion irradiation was used to induce varying degrees of cross-linking of aromatic self-assembled monolayers supported on a gold substrate, followed by removal of the non-cross-linked regions by adhesion to a PMMA film [112]. This leaves the exposed (cross-linked) regions behind, presenting an alternative to the conventional methods for carbon nanomembrane production in which cross-linking is induced by electron or UV exposure. Figure 5e shows a carbon nanomembrane from the above reference, fabricated by the HIBL method.

Finally, the neon ion beam of the HIM has been employed for resist-based lithography as well [113]. In fact, this happened only a couple of years after the first HIM HIBL work. Winston et al. showed that using neon ion beam lithography (NIBL), 7 nm diameter lines with a half-pitch also of 7 nm could be fabricated in HSQ (Figure 5f), which is comparable with both high-end EBL and HIBL. For similar beam energies, an exposure efficiency in the NIBL case of approximately one thousand times greater than EBL, and ten times greater than HIBL, was determined.

### 5 Direct-write lithography/milling

Direct-write ion beam lithography is a resistless technique in which patterning is achieved by direct milling with the focused ion beam (FIB). Due to helium ion implantation effects, the optimum sample geometry for direct-write helium FIB lithography is a free-standing film of thickness less than the penetration depth of the ions in the material. For such thin samples, helium implantation is largely avoided, because most of the ions pass right through. Furthermore, since there is minimal lateral scatter over such short distances and no backscatter from an underlying substrate, the sample can be milled with minimal beam-broadening effects and thus the narrowest cuts can be obtained. However, various strategies have also been developed to enable helium FIB milling of bulk samples that mitigate ion implantation effects, either by increasing the milling rate and thus decreasing the total dose, for example, by gas-assisted milling, or by using substrates that allow out-diffusion of the implanted ions. Another strategy involves the pre-milling of coarser features with the gallium FIB and/or neon FIB allowing the final precision cuts to be performed with the helium FIB at lower total dose, since by that stage less material has to be removed. For certain applications requiring precise efficient mills without gallium contamination, neon FIB for the final (or only) milling step is employed.

#### 2D and 1D materials

**Figure 6:**
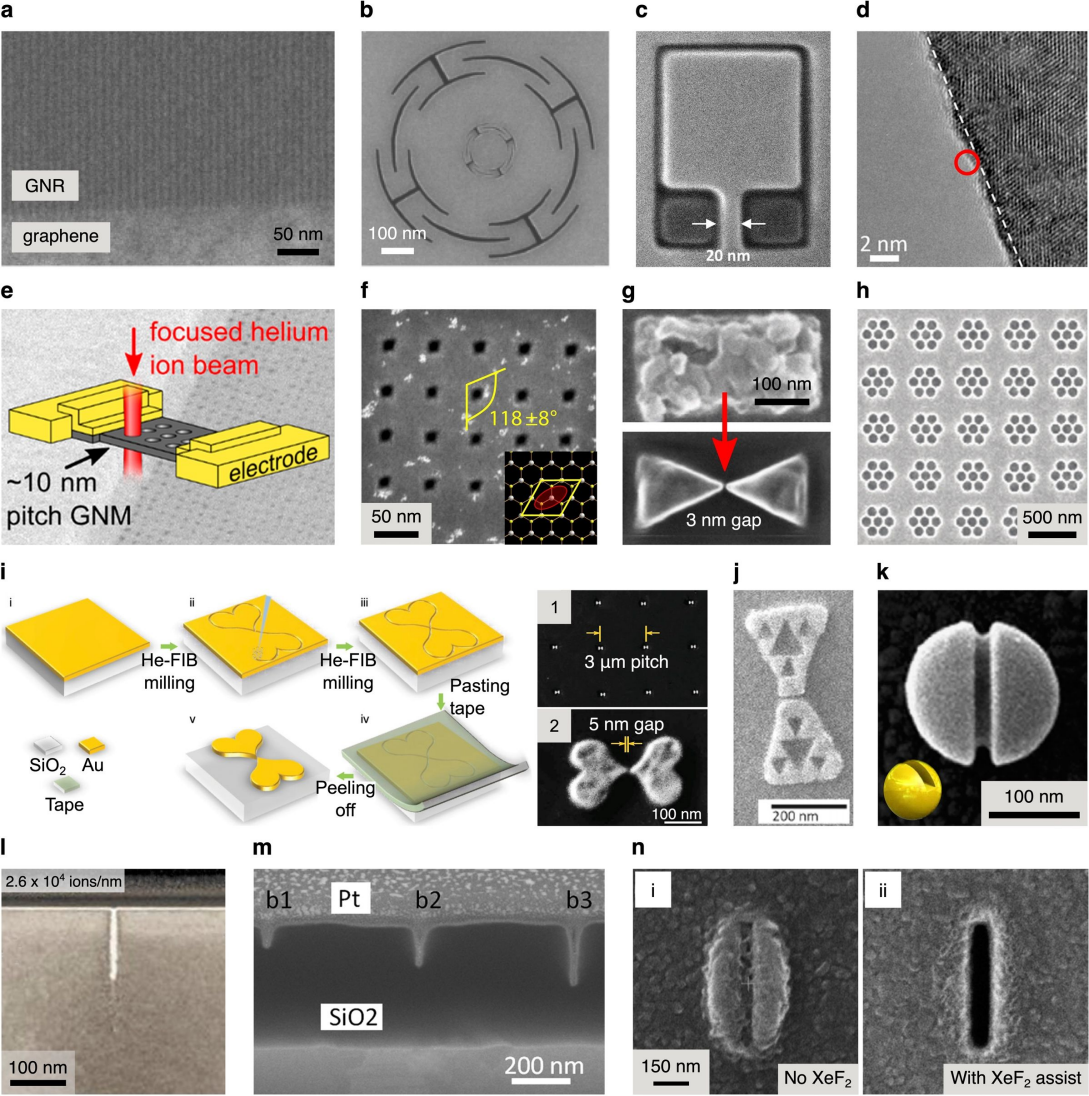
Examples of helium and neon FIB milling. **(a)** Graphene nanoribbons (GNR) fabricated by helium FIB milling of graphene on SiO_2_/Si. Adapted with permission from [132]. Copyright 2014 American Chemical Society. **(b)** Nanomechanical diaphragm structure (nested) fabricated by helium FIB milling of a free-standing graphene flake. Adapted with permission from [134]. Copyright 2011 IEEE. **(c)** Graphene pad structures fabricated by helium FIB milling of graphene on SiO_2_, imaged using the HIM: Dark contrast indicates pads that are electrically isolated; a 20 nm wide conductive strip connects the large pad at the top. Adapted from [135]. Copyright 2015 Iberi et al., licensed under a Creative Commons Attribution 4.0 International License, http://creativecommons.org/licenses/by/4.0/. **(d)** TEM image of a helium FIB-milled edge of a free-standing MoS_2_ flake: The dashed line indicates the extent of amorphization; the red circle represents the size of the helium ion beam used. Adapted with permission from [25]. Copyright 2015 American Chemical Society. **(e)** Free-standing graphene nanomesh (GNM) fabricated by helium FIB milling to demonstrate quantum confinement. Adapted with permission from [136]. Copyright 2018 American Chemical Society. **(f)** Rhombus-shaped nanopores in free-standing MoS_2_ fabricated using helium FIB milling implementing an elliptical beam shape. Adapted from [137], Deng et al., Nano-patterning of a monolayer molybdenum disulfide with sub-nanometer helium ion beam: considering its shape, size and damage, Nanotechnology, Vol. 31(34), 345302, first published 12 June 2020, Copyright © 2020 IOP Publishing Ltd. Reproduced with permission. All rights reserved. **(g)** Aluminum bow-tie plasmonic antenna on glass fabricated by helium FIB milling. Adapted with permission from [138]. Copyright 2018 American Chemical Society. **(h)** Plasmonic heptamer nanohole arrays in a gold film on SiO_2_ fabricated by helium FIB milling. Adapted from [139]. Copyright 2019 Hahn et al., licensed under a Creative Commons Attribution 4.0 International License, https://creativecommons.org/licenses/by/4.0/. **(i)** Schematic of the sketch-and-peel method using helium FIB milling to outline the desired shapes in a gold film on SiO_2_: (1) SEM image of an array of heart-shaped plasmonic dimer structures, (2) individual dimer. Adapted from [140]. Copyright © 2020 WILEY-VCH Verlag GmbH & Co. KGaA, Weinheim. Used with permission from Chen et al., Topology Optimization-Based Inverse Design of Plasmonic Nanodimer with Maximum Near-Field Enhancement, Advanced Functional Materials, John Wiley and Sons. **(j)** Gold bow-tie plasmonic antenna with internal fractal structure on glass fabricated by helium FIB milling of an EBL bow-tie pre-structure. Adapted from [141], Seitl et al., Miniaturized fractal optical nanoantennas defined by focused helium ion beam milling, Nanotechnology, Vol. 31(7), 075301, first published 14 November 2019, Copyright © 2019 IOP Publishing Ltd. Reproduced with permission. All rights reserved. **(k)** 3D plasmonic split-ball resonator fabricated by helium FIB milling of a silver nanosphere. Adapted by permission from Springer Nature, [142], Nature Communications, Split-ball resonator as a three-dimensional analogue of planar split-rings, Kuznetsov et al. Copyright 2014, Nature Publishing Group, a division of Macmillan Publishers Limited. All Rights Reserved. **(l)** Cross-sectional TEM analysis of a helium FIB-milled via in bulk silicon. Adapted with permission from [75]. Copyright 2014 American Vacuum Society. **(m)** Cross-sectional TEM analysis of neon FIB vias milled into SiO_2_. Adapted with permission from [143]. Copyright 2011 American Vacuum Society. **(n)** Helium FIB-milled slots in a titanium film (top views): (i) without, and (ii) with XeF_2_ gas assist. Adapted with permission from [144]. Copyright 2016 American Chemical Society. The content of (a), (b), (d), (e), (f), (g), (i), (j), (k), (l), (m) and (n) is not subject to CC BY 4.0.

In early helium FIB milling experiments, precise patterning of nanoribbons in a suspended graphene sheet was demonstrated, with nanoribbon widths down 5 nm achieved [131]. Graphene nanoribbons are of great interest, since they allow researchers to engineer a bandgap into the material due to quantum confinement effects. Furthermore, by creating the nanoribbons by direct milling as opposed to using a resist, resist residues and the resulting graphene ripples can be avoided. In work by Abbas et al., helium FIB milling of graphene on an SiO_2_/Si substrate was able to produce graphene nanoribbon arrays that indeed demonstrated semiconducting behavior [132]. An array from this work comprising 5 nm wide high-aspect-ratio nanoribbons closely packed with a half-pitch of 5 nm is shown in Figure 6a. The authors also investigated the potential use of these graphene nanoribbon arrays for chemical sensing, demonstrating exceptional sensitivity to NO_2_. In another study, helium FIB milling of graphene on SiO_2_/Si was used in combination with EBL to fabricate graphene-based quantum dot devices [133].

Returning our attention to free-standing graphene, helium FIB milling of such samples has also been used to fabricate nanomechanical diaphragm structures [51,134]. An example is shown in Figure 6b, where a set of curved and straight lines (with sub-10 nm linewidths) were helium FIB-milled into the graphene to form a radial arrangement of flexure beams. For further reading, an experimental and theoretical study of helium (and gallium) ion bombardment of free-standing graphene predicting the sputter yields for atomically thin targets can be found in [145]. And a further study of the dynamics of milling free-standing graphene with the helium FIB can be found in [146].

Samples typically need to be imaged with the HIM before milling commences (unless working “blind” with the aid of alignment markers). Therefore, it is important to establish safe imaging doses that will avoid the generation of unwanted defects in the material. Early studies by Fox et al. used Raman spectroscopy to investigate helium ion-induced defect densities in graphene and suggested a safe imaging dose of 10^13^ ions/cm^2^ for this material [147]. This equates to either an image recorded at low magnification, or a high magnification image acquired with a low signal-to-noise ratio. The authors also noted enhanced damage for supported graphene compared to free-standing, attributed to the interactions of backscattered ions and sputtered atoms from the substrate. However, damage is relative and the threshold doses to be applied depend on the particular experiment proposed. For example, elsewhere, irradiation of narrow stripe patterns into supported graphene using doses of 10^13^ ions/cm^2^ was shown to create enough disorder to induce a transition from metallic to insulator behavior [148]. A possible strategy to mitigate the beam damage of graphene in helium FIB milling applications involves encapsulating the graphene between flakes of hBN [149]. Here, Nanda et al. showed that defects induced by the helium ions in the sandwiched graphene sheet can heal by self-annealing and nitrogen doping. The efficacy of the technique was further investigated by the same group in an experiment in which encapsulated graphene sheets on an SiO_2_/Si substrate were milled with the helium FIB to create nanoribbons [150]. While defects and disorder in the nanoribbons beyond the patterned areas were still detected, the authors noted that the encapsulation method did prevent the surface contamination effects that readily manifest when milling unprotected graphene samples.

In further studies of helium (and neon) FIB milling of graphene on SiO_2_ substrates by Iberi et al., isolated graphene pad structures were created using perimeter line mills to define the pad structures and thus separate them from the surrounding graphene [135,151]. Here it was found that the secondary electron contrast upon imaging the pad structures with the ion beam enabled the in situ determination of whether or not an electrically isolated graphene pad had been obtained. As soon as the pad is isolated, dark contrast from the pad is observed, indicative of charging of the now non-grounded graphene on the insulating substrate. The authors demonstrated how the technique can be used to create narrow conducting graphene strips on the insulating substrate, gauging the preservation of the conductive behavior of the strips based on the contrast mechanism described above (see also Figure 6c). Effects limiting the minimum feature sizes achievable, including the ion beam profile and ion backscatter from the substrate, were discussed.

While most helium FIB milling of 2D materials has focused on graphene, a few studies have also focused on other 2D materials. For example, Fox et al. fabricated nanoribbons with diameters down to 7 nm in free-standing few-layer MoS_2_ [25]. High-resolution analysis of the nanoribbons by TEM revealed that sharp cuts with amorphous edges of thickness less than 1 nm could be achieved (Figure 6d). This damage layer correlated closely with the probe size of the helium ion beam used in the experiments (marked with the red circle in the figure) and is a good demonstration of what is possible when substrate backscatter effects are excluded through the use of a free-standing geometry. Helium FIB milling of nanoribbons into free-standing hBN has also been demonstrated [152], as well as the milling of high-quality grating structures into supported hBN flakes [153]. In the latter case for the supported material, neon FIB milling was shown to be advantageous due to the higher sputter yield while retaining a high-precision milling capability.

Helium FIB milling has also been applied to materials that could be classified as being 1D. For example, helium FIB milling has been used to create 3 nm nanogaps in metallic single-walled carbon nanotubes [154]. The nanotubes were supported on an SiO_2_/Si substrate and the nanogaps that were milled divided individual carbon nanotubes to form two closely spaced electrodes, which were used to probe individual organic molecules. In addition, linear arrays of holes have been helium FIB-milled along the lengths of free-standing amorphous SiCN nanobeams [155]. This work demonstrated the ability to tune the resonant behavior of nanomechanical resonators using a simple FIB-based post-fabrication method.

#### Nanopores

The fabrication of nanopores in thin membranes (free-standing, typically silicon nitride) is a further area that has benefited from the high milling precision of the helium FIB. Solid-state nanopores are central to pore-based biosensing applications, including DNA sequencing, in which the flow of ion current is monitored as the biomolecule passes through the pore. The size of the pore must be commensurate with the size of the biomolecule to be detected; double-stranded DNA has a width of ca. 2 nm. To be commercially viable, high-throughput methods for the fabrication of nanopores with precisely tuned diameters is key, and neither the gallium FIB nor electron beam-based "sculpting" techniques meet this need, since they are multi-step processes involving a final pore-closing routine to obtain the final diameters desired. In contrast, the helium FIB is able to routinely fabricate arrays of precise nanopores with diameters down to 4 nm in a one-step process [156], which has garnered significant interest in the field. Hall et al. have also reported the fabrication of individual nanopores in ca. 40 nm thick silicon nitride membranes with diameters down to 2.5 nm [157]. The pores are milled in spot mode (constant dwell on a single point) and the size of the pore is controlled by the irradiation dose. Through the implementation of automated stage movement and milling routines, patterning over large areas with high accuracy can be achieved [158]. A review of solid-state nanopore fabrication by helium FIB milling for the detection and analysis of biomolecules can be found in [159]. In closely related work, the translocation of virus particles (rod-shaped tobacco mosaic virus) through solid-state helium FIB nanopores has also been reported [160].

In order to monitor the thinning of the silicon nitride membrane in situ, and allow for sensitive endpoint detection, the transmitted ion current can be measured [161]. Tracking of the transmitted ion current has also been used for controlled thinning of larger areas of the silicon nitride membrane, followed by pore drilling with the helium FIB in spot mode in the normal fashion [162], or pore fabrication via electric field-driven controlled breakdown in the thinned regions [163]. The advantage of pre-thinning is the enhanced signal-to-noise ratio that is observed for translocation through nanopores in thinner membranes. Following the selective pre-thinning method described, this enhanced performance can be achieved while at the same time retaining the robustness of the device afforded by the overall thicker membrane. Measurement of the transmitted ion current during pore milling has also been used to track the growth behavior of larger pores, providing a convenient means to measure the point-spread function of the helium ion beam [164–165].

An important factor to take into account when performing any helium FIB nanofabrication (or even imaging, for that matter), is the potential detrimental effect of hydrocarbon contamination. For example, in work by Emmrich et al. using silicon nitride membranes exposed to differing degrees of plasma treatment prior to milling, it was found that the degree of hydrocarbon contamination remaining on the surface affected the subsequent milling of circle patterns when using different concentric scan strategies [164]. When milling a concentric circular pattern starting from the outside and moving in, hydrocarbon contamination was found to follow the path of the beam, limiting the size of the final milled pattern, whereas when the milling started from the center, hydrocarbons were prevented from diffusing inwards enabling the mill process to proceed largely unaffected. For the fully cleaned membranes, the scan strategy had no effect on the final pore size, since the hydrocarbon effect was no longer at play.

Apart from silicon nitride, helium FIB milling has also been used to create nanopores in other free-standing membranes, with minimum pore diameter records as follows: 1.3 nm diameter in a 1 nm thick carbon nanomembrane [164], 2.6 nm diameter in single-layer graphene [145], 4 nm diameter in few-layer and monolayer hBN [152,166], and 1.3 nm diameter in monolayer MoS_2_ [137]. Ion conductance and DNA translocation through helium FIB-milled graphene nanopores has been reported [167], as has the fabrication of suspended graphene nanomesh structures (3–4 nm pores on a pitch of ≤10 nm, Figure 6e) that demonstrated bandgap opening by quantum confinement [136]. In follow-up work to the latter, the size of the bandgap was tuned by varying the porosity of the nanomesh through adjusting the pitch of the periodic nanopore array [168].

In recent work on free-standing MoS_2_, another interesting phenomenon was observed, namely that by milling with a slightly stigmated beam (again, in spot mode), rhombus-shaped nanopores could be obtained with a geometry following that of the hexagonal MoS_2_ lattice [137] (Figure 6f). By helium FIB milling sub-10 nm pores into hBN and growing graphene into the voids, stable graphene quantum dot arrays have also been produced [166]. And when milling larger pores of 250 nm diameter into a 100 nm gold film on a glass substrate, the formation of “volcano-shaped” nanopores has been observed [169]. The raised perimeters around the pores may result from swelling of the gold, since helium ions have a relatively short stopping distance in this material, with possible contributions from the redeposition of sputtered atoms, or irradiation-induced mass transport.

The technically most challenging nanopores to fabricate are atomically precise ones consisting of just a few ejected atoms from a 2D material. Exploratory studies using both helium and neon ion irradiation in the HIM have been conducted in this area. For example, in work by Yoon et al., 500 nm square fields of free-standing graphene were irradiated with helium/neon ions by rastering the beam to achieve a uniform areal dose and subsequently the samples were annealed to stabilize the defects before inspection by high-resolution STEM [170]. Coupled with analysis based on atomistic simulations it was found that in the case of helium ion irradiation, vacancy defects involving local bond rearrangements were favored, whereas for neon ion irradiation, monovacancy defects were preferred. It was thus concluded that in order to transfer sufficient energy to efficiently displace carbon atoms from the lattice, irradiation with the higher mass ion (neon) is required. This work is directly related to the defect engineering studies described in Section 1, where helium ion irradiation of 2D materials was also used to create vacancies that were similarly investigated by high-resolution STEM [26,29–30]. Further work in this area has used Raman spectroscopy in combination with a model for defect activation to investigate the size and distribution of vacancy defects formed by broad-area illumination, using helium/neon ions and free-standing/supported monolayer graphene/MoS_2_ [171]. Very recently, the fabrication of atomic-scale nanopores in bilayer graphene for selective gas permeation was reported using a combined approach of helium ion bombardment to nucleate the defects followed by treatment in hydrogen plasma to expand the pores to the desired size [172].

A further interesting application involving helium FIB nanopores is the milling of holes into a thin layer of SiO_2_ on a silicon substrate to create dot arrays of exposed silicon that act as nucleation sites for semiconductor nanowire growth by molecular beam epitaxy [173].

#### Plasmonic structures

The precise milling capability of the helium FIB has also been used for the fabrication of various plasmonic structures, for which smaller gap sizes are critical in order to maximize the local field enhancement. For example, plasmonic dipole antenna structures have been fabricated whereby 20 nm thick gold islands (fabricated by EBL) on borosilicate glass were divided using a helium FIB cut, achieving gap sizes down to 3.5 nm [174]. Similarly, precise helium FIB thinning of a narrow band across the center of individual gold islands, followed by completely milling away the thinned material from the sides to leave a constriction in the middle, has been used to fabricate thin and narrow conducting bridges (bridge heights of a few nanometers and widths down to 5 nm) that can be used to modulate the plasmonic behavior of the antenna [175]. In a further study, helium FIB milling was used to create a ca. 5 nm gap in the elbow of L-shaped gold nanoantennae [176].

The fabrication of high-quality bow-tie antennae using the helium FIB has also been demonstrated. For example, in work by Kollmann et al., 30 nm gold on borosilicate was first milled using the gallium FIB to create a fused antenna structure of opposing triangular shapes, after which the helium FIB was used to create a sharp cut in the center giving a gap size of 6 nm [177]. The bow-tie structures fabricated in this manner had vastly improved non-linear performance due to the small gap with sharp sidewalls achieved by the helium FIB. The use of the helium FIB instead of the gallium FIB to fabricate the nanogap also means that implanted metallic residue at the milled interface is avoided, which would otherwise change the dielectric function of the material and degrade the plasmonic performance of the antenna. A similar fabrication of bow-tie structures in aluminum thin films has been demonstrated by Simeone et al., in this case achieving gap sizes down to 3 nm using the helium FIB to mill the entire structure into aluminum pads prepared by EBL [138] (Figure 6g). Here, a favorable smoothing of the surface of the evaporated aluminum during the helium FIB fabrication process was also observed, the underlying mechanism of which warrants further study.

Moving to other types of plasmonic structures, helium FIB milling of high-quality coaxial plasmonic resonators has been demonstrated [178]. In this work, Melli et al. used the helium FIB to mill 100 nm thick gold on fused silica to fabricate a plasmonic ring structure comprising a circular gap of diameter 200 nm and width 8 nm, which demonstrated an optical performance that matched theoretical predictions. The authors credited the high performance to the small gap size, vertical side walls, minimal edge rounding, and flat central mesa enabled by the helium FIB approach. In comparison, gallium FIB milling produced gap widths larger than 20 nm, V-shaped side walls, rounded edges and a perturbed top surface. By optimizing the gold film itself to enable more uniform milling rates, for example, by increasing the grain sizes in polycrystalline films by annealing [179–180], or by using single-crystal gold [181], further improvements in both gap size and reproducibility can be achieved. Additional plasmonic structures that have been fabricated by helium FIB milling include trimeric assemblies of resonant nanoapertures [182], arrays of nanostructured radial resonant apertures [183], and plasmonic heptamer nanohole arrays [139] (Figure 6h), all in gold films, as well as multiresonant plasmonic nanoslit cavities [184] and plasmonic tetramer nanoantennae [51] in crystalline gold platelets. In the latter study, an advanced beam patterning toolbox with beam path optimization was recently introduced [51].

A novel strategy for the fabrication of large assemblies of plasmonic structures is the so-called “sketch and peel” method [185–186]. First developed by Chen et al. for EBL, it was then extended to FIB milling, thus circumventing the need for a resist and enabling more flexibility in terms of the substrate. In the FIB method, ion milling is used to outline, or “sketch” shapes into a supported gold film, after which the unwanted portions of the film are removed by selective peeling. The explanation for the selective peel is proposed to be redeposition of sputtered material onto the sidewalls of the cuts, which protects the outlined shapes from being removed with the rest of the film during the peeling process. The significant advantage of this technique is that large assemblies can be fabricated rapidly, since only the outlines need to be milled, rather that having to mill away all of the gold film around each desired structure. For helium FIB milling, the high speed of the sketch-and-peel approach is particularly compelling, due to the inherently low sputter yield of the light ions. Furthermore, for all the areas that are not patterned by the beam, beam damage to the underlying substrate is avoided. Compared with the gallium FIB, sketch-and-peel using the helium FIB enables finer cuts and, in the first demonstrations, the fabrication of arrays of plasmonic dimer structures with a gap size of 15 nm was achieved [186]. Subsequent iterations of the method routinely report gap sizes of 5 nm [180–181], including the fabrication of heart-shaped nanodimers defined using a shared boundary in the center to create the nanogap as opposed to milling the dimer outline and gap separately [140] (Figure 6i). To further increase the efficiency of the process, a combination of gallium and helium FIB milling can be applied, the former for the coarser outlines and the latter for the fine cuts forming the nanogaps [180,187]. The advantage of using single-crystal gold to ensure uniform sputter rates, and thus ultimately enable milling of narrower cuts with enhanced reproducibility, has also been demonstrated for the sketch-and-peel method [181]. In the latter, Laible et al. also investigated the fabrication of gold pre-stuctures by EBL and argon ion milling (rectangles or connected bow-tie shapes) as another means to reduce the milling time for large assemblies, whereby the final nanostructures themselves were defined using optimized helium FIB milling strategies based on multidirectional scans. In follow-up work by the same group, bow-tie nanoantennae with fractal internal geometries were fabricated, using bow-tie pre-structures prepared by EBL that were then milled with the helium FIB to accurately define the nanogaps and the internal fractal patterns [141] (Figure 6j).

3D plasmonic structures with tunable magnetic response at optical frequencies have also been fabricated by helium FIB milling [142]. In this work, spherical nanoparticles of gold/silver were fashioned with a precise nanoscale cut using the helium FIB, creating “split-ball” resonators with nanometer control over the width and depth of the cut (Figure 6k). In another 3D example, the helium FIB has been used to mill nanoscale apertures into the center of hollow pyramidal [188] and hemispherical [189] gold microstructures for sensing applications via local electric field enhancement. 3D plasmonic gold tips that were pre-milled with the gallium FIB and then finely shaped with the helium FIB have also been produced [190].

#### Bulk samples: semiconductor industry applications and further next-generation devices

As mentioned previously, the ideal sample for helium FIB milling is a free-standing material that is thinner than the stopping distance of the helium ions, such that most pass through and thus cannot accumulate. However, suspended membranes are not always the desired geometry for devices, and indeed a number of the examples already given were for thin materials supported on bulk substrates. For FIB applications in the semiconductor industry (circuit edit, failure analysis, mask repair, and device prototyping), the samples of prime concern are bulk materials, and thus bulk effects must be considered and mitigated. In fact, for semiconductor applications and for the fabrication of next-generation devices on bulk substrates, the neon FIB is of particular interest, due to the higher sputter yield and reduced penetration depth of the heavier ions.

Certain substrates are less susceptible to the swelling that can occur under helium ion irradiation, since they allow the helium to diffuse out. These favorable substrates are non-crystalline and a key one is amorphous SiO_2_, for example, in the form of several hundred nanometers of amorphous SiO_2_ on a silicon wafer, or as a bulk glass substrate. If such a substrate can be selected, the full advantages of helium FIB milling to create narrow cuts with high aspect ratio can be realized. For example, in early work, 10 nm wide vias with an aspect ratio of up to 1:12.5 in a supported gold layer were reported [191]. Using gallium FIB milling, such high aspect ratios are unobtainable, due to the significant taper angle of the sidewalls that give gallium FIB vias their characteristic V-shape, with aspect ratios rarely above 1:5. The strongly tapered side walls of gallium FIB vias largely result from redeposition, and while there is also redeposition in helium FIB vias, the sputter yield under glancing incidence for helium ions is much more strongly peaked compared to gallium ions, thus significantly reducing the taper effect [192]. Further demonstrating the small taper angle for helium FIB milling, through-holes in a (free-standing) 100 nm thick gold film with diameters of 5 nm and, thus, aspect ratios of 1:20 have also been reported [193].

Significant differences between the beam–sample interaction profiles for thin-film compared to bulk sample geometries have been revealed by Tan et al. through cross-sectional TEM analysis of helium FIB line mills of varying dose in suspended silicon membranes compared to bulk single-crystal silicon [75]. For the bulk sample, a via starts to form, but at high dose closes again as a result of damage accumulation and volume expansion due to the implanted helium. Eventually, significant amorphization occurs within a characteristic teardrop-shaped volume. In contrast, in the membrane case, forward-sputtering from the back of the membrane dominates and the overall sputter yield is much higher, with minimal helium implantation, thus enabling the milling of narrow high-aspect-ratio vias. As the milling progresses and the membrane thins, the ratio of back- to forward sputtering changes, eventually giving rise to an upturned V-shaped profile.

However, as mentioned, it is the bulk sample that is of concern in the semiconductor industry. Although sharp vias can be obtained in bulk silicon using the helium FIB when selecting the appropriate dose, as shown in Figure 6l [75], helium ions are still implanted at the base. For applications such as circuit edit and mask repair, the deep penetration depth of helium ions and associated damage effects (amorphization, nanobubble formation, and swelling) cannot be tolerated. Moreover, a higher sputter efficiency is needed so that larger volumes of material can be removed. Here, the neon FIB is a favorable alternative, since it has a shallower implant depth than helium and a higher sputter yield, as first shown by Tan, Livengood, and co-authors, and later by others [15,17,100,143,194]. In fact, the sputter yield for neon increases for decreasing beam energy (which is the reverse of what happens for gallium). Thus, it is actually more favorable to operate at lower neon beam energies (e.g., 10 keV), which also reduces the penetration depth and amount of subsurface damage [15,195]. This is important, because as with helium ion irradiation, neon nanobubbles can still form and the associated swelling can still occur [15,17,100,195–198].

By using the neon FIB in place of gallium FIB, the detrimental contamination of semiconductor materials with gallium is avoided. Further, as recently demonstrated for 10 keV neon FIB milling of the group-III–V semiconductor GaAs, trenches with a smooth surface at the base can be produced (i.e., no porosity due to neon nanobubbles), which also do not exhibit the gallium-rich droplets that occur for gallium FIB milling as a result of preferential sputtering of the group-V element [195]. It should be noted, however, that the milling behavior of the group-III–V semiconductors varies, with different morphological changes observed depending on the particular material in question. For example, in experiments on GaSb milled using neon ions of the same energy and at the same dose as in the previous study, surface porosity was induced, whereas at slightly lower doses, the formation of gallium-rich nanodots on the GaSb surface occurred [198]. Thus, in addition to creating smooth surfaces, neon FIB milling can also be used for selective surface nanostructuring.

Neon FIB vias with relatively high aspect ratios (up to ca. 8:1), compared with their gallium FIB counterparts, have been demonstrated, although they are similarly limited by a V-shaped profile [17,143,199] (Figure 6m). 3D Monte Carlo simulations of the evolution of the neon FIB via profiles have been performed and find close agreement with the profiles determined experimentally, explaining the sidewall tapering as resulting from an increase in redeposition rate and a decrease in effective sputter rate with depth, correlating with the decreasing open angle of the via [196,200–201]. Recent refinements to the Monte Carlo code by Mahady et al. have revealed that in order to accurately predict the width of the neon FIB vias, an effective beam profile needs to be used as the input, rather than the measured one. This is because artifacts such as platform vibrations seem to be broadening the mills, resulting in simulated via widths that are narrower than the real ones [202].

The favorable sputter yield of neon and the fact that the implanted ions are not metallic has also been put to use for the fabrication of superconducting nanowires on bulk substrates, where selected regions of Nb and NbN films were milled away to form constrictions of the desired dimensions [203–205]. Furthermore, neon FIB milling has been used to investigate the effect of miniaturization on the switching currents of magnetic tunnel junction devices by successively reducing the device size using neon FIB cuts into the sub-10 nm range [206].

#### Minimizing subsurface damage

Apart from working with thin free-standing samples or selecting substrates that are less susceptible to swelling, one approach to reduce substrate swelling is to heat the sample during the milling. This encourages out-diffusion of the implanted helium before it can accumulate and also promotes annealing of ion-induced defects. Techniques using an in situ MEMS-based heater [207] and localized in situ pulsed laser-based heating [208] have been developed. In the latter, Stanford et al. showed that around 90% of the implanted helium can be driven out using the laser-assist process, and that peripheral damage when milling graphene on an SiO_2_ substrate can also be mitigated. Pulsed laser assist during helium FIB milling has also been shown to be an effective strategy to mitigate the ion beam-induced deposition of carbon from residual hydrocarbon species that can otherwise compete with the milling process [102].

Another approach to minimize subsurface damage is to increase the milling rate, so that the total ion dose (and hence total amount of implanted helium) required to mill a given feature is significantly reduced. To achieve this, gas-assisted etching can be employed. This involves delivering a gaseous precursor to the region of the sample being milled, just as one does in FIB-induced deposition (see Section 6), except here, a gas is chosen that will generate radicals upon dissociation of its molecules that then form volatile reaction products with atoms from the substrate material, thus enhancing the rate of their removal. Using this method, XeF_2_-assisted helium FIB milling of a 200 nm thick titanium film on a 30 nm silicon nitride membrane was demonstrated [144]. The authors showed that by increasing the milling rate, the amount of subsurface damage could be minimized to such an extent that swelling was essentially eliminated (Figure 6n). Gas-assisted helium FIB milling using XeF_2_ has also been demonstrated for the fabrication of WSe_2_ nanoribbons on SiO_2_ substrates, with a fivefold enhancement of the mill rate reported [209]. Yet, it was noted that defects were still generated up to 120 nm from the milled edges due to backscattered ions and recoiled atoms from the substrate. As for the neon FIB, Monte Carlo simulations together with experiments have been used to investigate XeF_2_-assisted neon FIB milling of vias in SiO_2_ [210]. Prior to this work, experimental studies of the assist process for neon FIB milling of larger areas of an SiO_2_ target had also been performed [199], and more recently, XeF_2_-assisted helium and neon FIB milling of TiN thin films [211] and resist patterns [194] has been investigated.

Laser-assisted milling can also be combined with gas-assisted milling to increase milling rates even further. This is because thermal effects increase the diffusion rates of the XeF_2_ precursor molecules and of the reaction by-products. Using this combined approach, Stanford et al. showed that approximately a ninefold enhancement in helium FIB milling rates compared to non-assisted milling can be achieved [144].

Finally, using a method without any beam assist, it has been shown that the sample can be tilted relative to the beam normal in order to favorably shift and foreshorten the volume of the beam–sample interaction [212]. In this work by Hang et al., 30 keV helium ions were used to mill lines into graphene supported on a substrate comprising SiO_2_(300 nm)/Si. Under normal incidence, significant substrate swelling occurred, which can be attributed to a significant proportion of the helium ions accumulating in the silicon. In contrast, by tilting the specimen from the horizontal position by 43°, swelling was avoided, since then most of the ions accumulated in the amorphous SiO_2_ layer, allowing the helium to diffuse away. However, through analysis by Raman spectroscopy, defects in the graphene lattice extending up to 300 nm away from the milled lines were detected, indicating damage caused by backscatter helium ions and possibly also recoiled substrate atoms.

#### Specimens for materials analysis

Another important application of FIB milling in general is the fabrication of electron-transparent specimens for materials analysis by TEM. The gallium FIB has been the workhorse instrument in this area. Yet, there are certain samples that are susceptible to artifacts from gallium ions. Hence, alternative ion species for FIB-based preparation are of interest. For example, aluminum and its alloys tend to become contaminated and embrittled as a result of the segregation of gallium along grain boundaries and interfaces. Also, semiconductor materials have been shown to develop gallium precipitates and droplets [213–214].

The use of the helium FIB [215] and neon FIB [197] to thin TEM lamellae has been investigated. In these studies, the lamellae were first pre-thinned using the gallium FIB and then the final thinning step was performed using helium/neon. Under glancing incidence, helium FIB milling was shown to reduce surface roughness, remove surface contamination, and produce thinner specimens with crystallinity preserved [215]. However, helium FIB milling is inherently slow due to the low sputter yield of the light ion. Therefore the neon FIB, with a higher sputter yield, while retaining the precision characteristics of the GFIS beam, is a lucrative alternative. By switching from gallium to neon milling at a judicious time point (i.e., while the central region of the lamella is still gallium-free), it has been shown that final TEM specimens that do not contain gallium can be obtained [197]. Another advantage of the neon FIB method is that during the final milling stage, the thickness of the lamella can be monitored accurately by end-on imaging using the neon ion beam. Compared with imaging with low-energy gallium ions (low-energy gallium is conventionally used for the final mill step), the thickness of the lamella can be visualized very clearly using the neon ion beam. However, although the neon FIB allows for more precise milling, it should be noted that beam-induced damage such as dislocations are still comparable to those resulting from the conventional gallium FIB method [197]. The application of neon FIB milling for the fabrication of tips for atom probe tomography, similarly limited by gallium-only milling for certain materials, is also underway [216].

FIB milling is also widely used to fabricate specimens for micro- and nanomechanical tests. Again, gallium contamination can be a concern, this time due to potential changes in the mechanical properties of the material. Thus here too, FIB species other than gallium are being explored. In this regard, helium FIB milling has been tested to create sharp notches for small-scale fracture toughness measurements [217]. Although sharp notches could be produced, a significant limitation is helium implantation into the material beneath the notch forming nanobubbles, which also affects the nanomechanical behavior of the material. Similarly, when using the helium FIB to prepare silicon nanopillars for in situ TEM nanomechanical compression testing, nanobubbles formed inside the pillars resulting in a decrease in the yield stress of the material [89]. In future work, gas-assisted etching might be used to increase sputter rates and thus decrease the total amount of ion implantation, as described earlier. Alternatively, sample geometries that in the milling direction are thinner than the helium ion stopping distance could be considered. Neon FIB milling is also a possibility. Indeed, in a study in which the neon ion beam was used to irradiate silicon nanopillars, it was found that by irradiating at elevated temperature, the material remained crystalline and controlled thinning by sputtering could be achieved [218]. In contrast, at room temperature, amorphization occurred and the nanopillars evolved into conical shapes due to viscous flow.

### 6 Gas-assisted deposition

FIB-induced deposition (FIBID) is a further area of direct-write nanofabrication benefiting from the high-resolution characteristics of the HIM, enabling the fabrication of structures with dimensions smaller than are possible using conventional gallium-based FIBID. Moreover, the deposition rates of helium FIBID have been found to approach those of focused electron-beam-induced deposition (FEBID), thus realizing a form of FIBID that delivers both high resolution and high rate. Similarly to resist-based HIBL, proximity effects are also minimized in helium FIBID. For a detailed review of early helium FIBID work, the reader is referred to [219].

#### Deposition rates, deposit dimensions, compositions and resistivities

In FIBID, a chemical precursor is introduced into the vacuum chamber, typically in the form of a gas injected via a needle placed close to the specimen. The precursor molecules are then dissociated into volatile and non-volatile fragments as a result of interactions with the primary ion beam, secondary electrons, and backscattered ions, with potential contributions also from excited atoms and thermal effects [220]. The non-volatile fragments are deposited at the locations scanned by the beam and the volatile fragments are pumped away.

The first studies of helium FIBID were reported by Sanford et al. and used a standard FIBID/FEBID platinum-based organometallic gaseous precursor to grow broad deposits in the micrometer size range [221]. Deposition rates of 0.2–0.6 μm^3^/nC were reported, comparable to those achieved by gallium FIBID (i.e., relatively fast). In terms of purity, percentage compositions of platinum of up to 20% were measured, comparable to those typically obtained by FEBID (i.e., fairly low). Yet later, Drezner et al. experimented with ca. 200 nm wide pillar deposits and demonstrated that by varying the beam current, higher purity deposits containing up to 40% platinum could be achieved, thus approaching the purity levels of gallium FIBID [222], without co-implanting gallium. In this particular work, it was found that decreasing the beam current decreased the deposition rate, resulting in shorter pillars with a higher platinum content. This was attributed to a higher availability of precursor when the growth rate was slower. A similar trend for the growth rate as a function of ion current was reported by others [223]. However, in Sanford’s earlier study [221], and in later work investigating the growth of much narrower pillars (described further below), the opposite was found, with decreasing current resulting in faster vertical growth rates [224]. This highlights the complex interplay of processes involved in FIBID and the important difference between operating in a reaction-limited versus a mass-limited regime. Interestingly, Drezner and co-authors also found that the platinum content varied along the length of their pillars, and was greatest near the top [222]. Amorphization and swelling of the substrate immediately beneath the deposit were also noted.

Soon after the first demonstration of helium FIBID for relatively large deposits, Chen et al. demonstrated the growth of platinum-based nanopillars by continuous illumination in spot mode, achieving diameters down to 36 nm, that is, approximately four times narrower than nanopillars grown by gallium FIBID and comparable to the minimum widths achieved by FEBID [224]. In early work, very tall nanopillars with aspect ratios up to 1:130 were also demonstrated [225], and more recently, aspect ratios of 1:200 have been reported [226]. An analytical model [227] and Monte Carlo simulations [224,228] were developed to investigate the deposition mechanism, concluding that pillar growth in the vertical direction (height) is due to the precursor dissociation reactions induced by the incident ions and the secondary electrons they generate, whereas lateral pillar growth (width), is predominantly caused by the interactions of scattered ions and their associated secondary electrons as they exit the sides of the pillar. Since the density of primary ions and their secondary electrons at the apex is much higher than that of the scattered ions and associated secondaries at the sides, the growth rate in the vertical direction is much faster and pillars with high aspect ratio can be obtained. Furthermore, since there is limited backscatter of ions from the substrate, proximity effects are minimized. For example, the halos that form around nanopillars grown by FEBID are not observed in the helium FIBID case. For constant ion dose it was found that decreasing the ion current resulted in the tallest and narrowest pillars, indicating a growth rate regime limited by the availability of precursor at the apex [224,227–228]. Subsequent work by Alkemade et al. investigated pulsed deposition, achieved by scanning an array of points in a cyclic fashion to grow a set of nanopillars in parallel (rather than growing individual nanopillars one by one, by continuous illumination of the same spot) [229]. It was found that a pulsed beam approach can alleviate depletion of the precursor at the apex, since the precursor supply is constantly refreshed. Following this approach, the authors demonstrated enhanced vertical growth rates for pulsed deposition, with the fastest rates achieved for the shortest dwell times.

**Figure 7:**
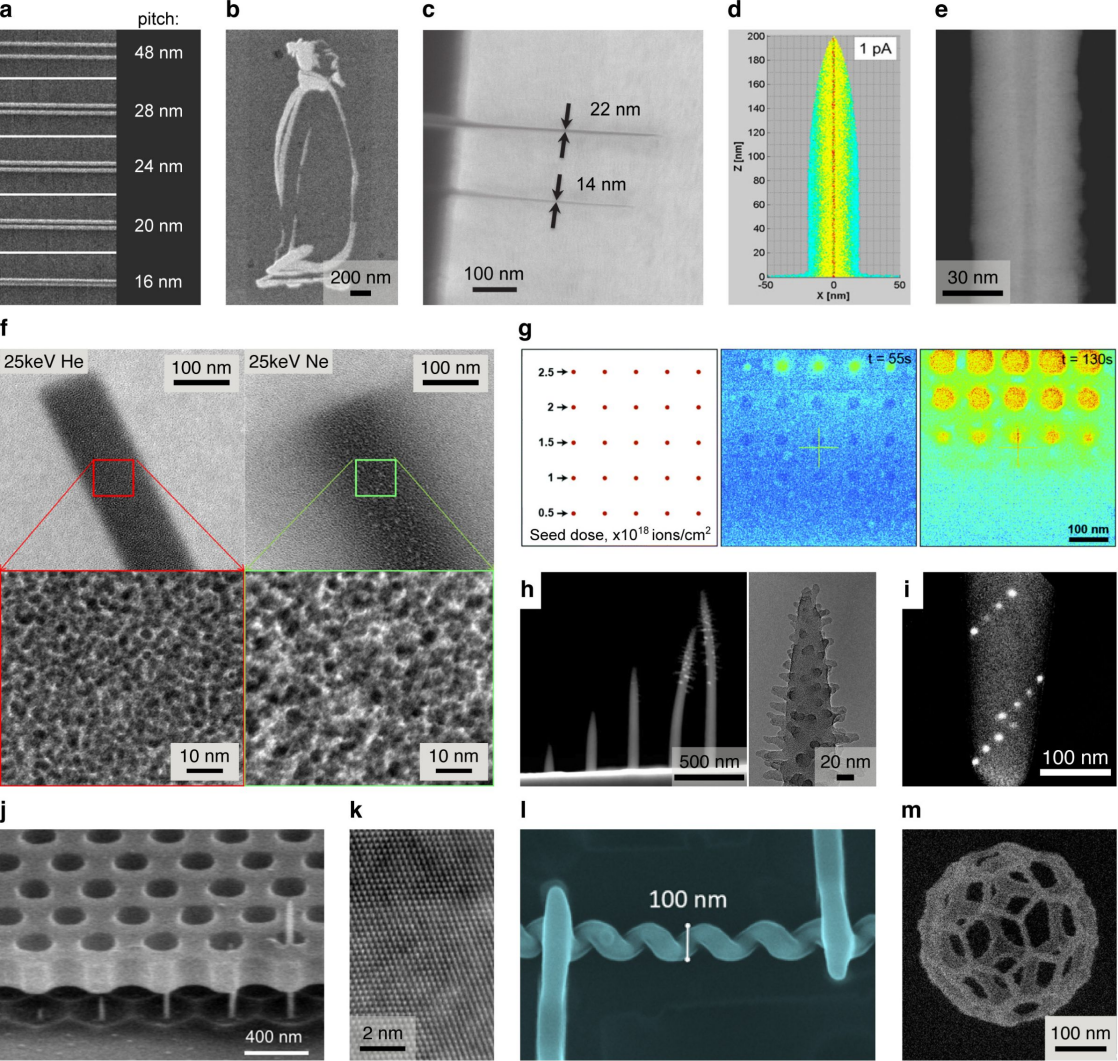
Examples of helium and neon FIBID. **(a)** Platinum-based line deposits fabricated using helium FIBID demonstrating a half-pitch down to 8 nm. Adapted with permission from Cambridge University Press, [232], Scipioni et al., A Design-of-Experiments Approach to Characterizing Beam-Induced Deposition in the Helium Ion Microscope, Microscopy Today, 19(3), 22-26. Copyright 2011 Microscopy Society of America. **(b)** Reproduction of a 13th century painting of a Chinese poet using helium FIBID with line widths down to 10 nm. Adapted from [235]. Copyright © 2012 Wiley Periodicals, Inc. Used with permission from Alkemade et al., Imaging and Nanofabrication With the Helium Ion Microscope of the Van Leeuwenhoek Laboratory in Delft, Scanning, John Wiley and Sons. **(c)** Horizontally grown platinum-based nanopillars using helium FIBID. Adapted with permission from [236]. Copyright 2015 American Vacuum Society. **(d)** Cross-section through a 3D Monte Carlo simulation of a helium FIBID nanopillar colorized according to the particles inducing the deposition: red (primary ions), yellow (secondary electrons - first generation), green (forward-scattered ions), cyan (secondary electrons - second generation). Adapted from [224], Chen et al., Nanopillar growth by focused helium ion-beam-induced deposition, Nanotechnology, Vol. 21(45), 455302, first published 14 October 2010. Copyright © 2010 IOP Publishing Ltd. Reproduced with permission. All rights reserved. **(e)** Hollow tungsten-based nanopillar fabricated using helium FIBID, imaged by STEM. Adapted with permission from [238]. Copyright 2013 American Vacuum Society. **(f)** TEM analysis of platinum-based in-plane nanowire deposits fabricated using helium FIBID (left) and neon FIBID (right). Adapted from [239], Wu et al., Synthesis of nanowires via helium and neon focused ion beam induced deposition with the gas field ion microscope, Nanotechnology, Vol. 24(17), 175302, first published 2 April 2013, Copyright © 2013 IOP Publishing Ltd. Reproduced with permission. All rights reserved. **(g)** Helium FIBID using a liquid precursor capped by a silicon nitride membrane: Nanoparticles are first seeded at pre-defined locations using point exposures, then the beam is rastered over the full area for controlled growth/imaging (colorized HIM images for two successive time points are shown). Adapted with permission of Royal Society of Chemistry, from [240], Building with ions: towards direct write of platinum nanostructures using in situ liquid cell helium ion microscopy, Ievlev et al., 9(35), Copyright 2017; permission conveyed through Copyright Clearance Center, Inc. **(h)** Branched nanopillars obtained by helium FIBID of an insulator precursor, imaged by (S)TEM. Adapted from [241]. Copyright 2021 Allen, licensed under a Creative Commons Attribution 4.0 International License, https://creativecommons.org/licenses/by/4.0/. **(i)** Tungsten-based nanodots fabricated using helium FIBID serving as alignment markers for electron tomography. Adapted from [242], Micron, 50, Hayashida et al., High-precision alignment of electron tomography tilt series using markers formed in helium-ion microscope, 29-34, Copyright (2013), with permission from Elsevier. **(j)** Local modulation of the dielectric properties of a photonic crystal with platinum-based nanopillars fabricated using helium FIBID. Adapted from [219] by permission from Springer Nature and with kind permission from the author P.F.A. Alkemade, Applied Physics A, Focused helium-ion-beam-induced deposition, Alkemade, P.F.A., and Miro, H. Copyright 2014 Springer-Verlag Berlin Heidelberg, Copyright P.F.A. Alkemade. **(k)** High-resolution STEM analysis of a portion of a tungsten-based nanopillar grown vertically by helium FIBID. Adapted from [243]. Copyright 2020 Córdoba et al., licensed under a Creative Commons Attribution 4.0 International License, https://creativecommons.org/licenses/by/4.0/. **(l)** Tungsten-based nanohelix fabricated using helium FIBID (with platinum contacts). Adapted with permission from [244]. Copyright 2019 American Chemical Society. Further permissions related to the material excerpted should be directed to the ACS. **(m)** Platinum-based deltahedron fabricated using helium FIBID. Adapted from [245]. Copyright 2020 Belianinov et al., licensed under a Creative Commons Attribution 4.0 International License, https://creativecommons.org/licenses/by/4.0/. The content of (a), (b), (c), (d), (e), (f), (g), (i), (j) and (l) is not subject to CC BY 4.0.

Since the width of the nanopillar is determined by lateral scattering, as described above, the nanopillar widths are greater than the beam diameter (pillar widths are typically 30–40 nm). And since the beam spreads in a conical fashion to a depth of around 300 nm, the tips of helium FIBID nanopillars are markedly cone-shaped [224,227,230]. This feature can be used to create smaller-diameter (ca. 10 nm) dot deposits using short dwell times, so that only the first part of the conical shape is formed [231]. Alternatively, the beam can be scanned horizontally across the surface to deposit lines. For example, pairs of lines with line widths down to 13 nm on a half-pitch down to 8 nm have been demonstrated, as shown in Figure 7a [232]. Individual lines with diameters down 10 nm have also been achieved [233–234], including in the free-form patterning of a miniaturized reproduction of a 13th century painting [235] (Figure 7b). With respect to the nanopillars, an effective method to reduce their diameter is to gradually move the beam laterally during the deposition, rather than hovering over the same point in spot mode. In this way, a nanopillar is grown horizontally rather than vertically, and since the sample volume beneath the beam interaction point is thus significantly reduced, there is much less broadening and narrower structures can be grown [236]. Following this approach, nanopillars with diameters down to 14 nm have been demonstrated (Figure 7c). In further regard to pillar widths, 3D Monte Carlo simulations have in fact predicted that neon FIBID nanopillars could have even smaller diameters than their helium FIBID counterparts, due to the smaller overall interaction volume for neon causing less broadening further down the pillar, and also due to the lower secondary electron emission yield [237]. Experimental investigations of this proposed effect for neon FIBID are still pending.

A unique feature of helium FIBID nanopillars is that they can have hollow cores. This is a direct consequence of the highly localized interaction of the helium ions, as illustrated by Monte Carlo simulations of helium FIBID nanopillar growth, see Figure 7d [224]. In the referenced figure we see a cross-section through a simulated helium FIBID nanopillar, color-coded according to the species that induced the deposition in a particular area. The central column, shown in red and just a nanometer or so wide, indicates the region where deposition is induced by the primary helium ions. However, deposition always competes with sputtering, and if the sputtering rate is high enough, then the central region of the pillar can be milled out as the nanopillar grows, creating a columnar void. Hollow helium FIBID nanopillars were first observed by Kohama et al., with the hollow cores visualized by STEM [238] (Figure 7e). In the case of hollow nanopillars grown on silicon substrates, the authors also noted subsurface damage in the form of swelling. They showed that this swelling can be minimized by increasing the growth rate of the nanopillar, since then the pillar can more quickly shield the material below. Interestingly, in the case of thin amorphous carbon substrates (about 300 nm thick), which did not exhibit such pronounced swelling, the growth of a smaller nanopillar “twin” on the back surface was observed in addition to the one on the top, with a columnar void in each [246].

The electrical resistivities of helium and neon FIBID deposits have also been investigated. For metallic deposits where high conductivity is desired, gallium FIBID is advantageous, since co-implanted gallium increases the metal content thus improving the conductivity. In contrast, FEBID generally results in lower metal contents, both due to the nature of the electron beam-based deposition reaction as well as due to the absence of a co-implanted metal species. In helium and neon FIBID experiments by Wu et al. using the standard platinum-based precursor, a number of nanowires were deposited and their resistivities were measured [239]. It was found that the helium FIBID nanowires had rather high room-temperature resistivities (i.e., low conductivity), similar to those obtained using FEBID. In contrast, the resistivities of the neon FIBID nanowires were found to be significantly lower. TEM elemental analysis showed that the platinum content of the nanowires was actually about the same in each case (15–16%); the observed difference was the microstructure. For the helium FIBID nanowires, small platinum nanocrystals (about 3 nm in diameter) in a carbon matrix were observed, whereas for the neon FIBID nanowires slightly coarser nanoparticles in the carbon matrix (about 4.5 nm in diameter) were present (Figure 7f). Thus, the difference in conductivities was attributed to the difference in platinum particle size, with the larger particle size in the neon case proposed to be due to the reduced range of those ions and thus higher density of energy deposition (neon ion stopping being dominated by nuclear scattering, as opposed to electronic scattering for helium).

Precursor choice also has a significant effect on the resistivity. For example, in work using a cobalt-based precursor, helium FIBID achieving high-purity low-resistivity metallic nanowires was demonstrated, with TEM analysis revealing cobalt nanocrystallites of ca. 6 nm diameter and negligible carbon content [233]. Crucially, this work showed that by appropriate choice of the precursor, both high-resolution and high-purity deposits can be obtained using the helium FIBID method. Very recently, differences in composition and resistivity for tungsten-based nanowires depending on whether the deposition was performed in-plane onto the substrate or vertically in a nanopillar geometry were highlighted, pointing to differences in the relative contributions of the various beam–sample interactions in each case [234].

The precursor also needs not be a gas. In work by Ievlev et al., helium FIBID using a liquid precursor has been demonstrated [240]. In this setup, an in situ liquid cell is filled with a liquid precursor and capped with a 50 nm thick silicon nitride window. The helium ion beam is focused onto the window at pre-defined locations to seed nanoparticles on the back side (i.e., in the liquid) and the ion beam is then rastered over the window for controlled growth of the deposits and imaging (Figure 7g). An advantage of this approach over the usual gaseous precursor method is that the purity of the deposits can be greatly enhanced. For example, using a K_2_PtCl_6_ liquid precursor, the researchers demonstrated platinum deposit purities of 95%.

As for the deposition of insulating materials, FIBID using noble gas beams has the natural advantage of being able to create insulating deposits without any trace of implanted metallic species. Indeed, helium and neon FIBID of pad structures using the precursor pentamethylcyclopentasiloxane has been shown to yield resistivities two to three orders of magnitude higher than those achieved by gallium FIBID [199]. Helium FIBID of nanopillars using an insulator precursor has also been shown to result in the formation of side branches on the main body of the pillar, attributed to a charging effect [192,241] (Figure 7h).

#### Applications of helium FIBID

Early on, the fabrication of nanodots by helium FIBID was demonstrated as a technique to obtain fiducial markers at pre-defined locations to be used to align image series for TEM tomography [231,242] (Figure 7i). The use of helium FIBID to pattern seed layers for subsequent growth by atomic layer deposition has also been explored [235,247]. However, the most active area of research using helium FIBID has been based on the fabrication of nanopillars. For example, platinum-based nanopillars have been used to modulate the dielectric constant of individual holes in a photonic crystal [219]. This was achieved by suspending the photonic crystal above a substrate and growing the nanopillars onto that substrate such that they protruded into the holes of the photonic material (Figure 7j). Helium FIBID nanopillars have also been grown onto atomic force microscopy (AFM) cantilevers to create sharp tips with high aspect ratios [248]. And the horizontal nanopillar growth method mentioned previously has been used to grow even narrower tips with a perpendicular bar shape at the end to create hammerhead probes for AFM [236].

The phenomenon of hollow helium FIBID nanopillars has been used by Córdoba et al. for the fabrication of superconducting hollow tungsten-based crystalline nanowires (tungsten contents of up to 70 atom %) [226]. The structures were grown vertically and then laid down onto substrates for four-point-probe magnetotransport measurements. The particular superconducting behavior of these hollow nanostructures, observed upon cooling to 6.4 K, has been attributed to the pinning of superconducting vortices along a single line in the core, creating a quasi-1D superconductor. A study of the effect of current and dose on the lateral dimensions of the hollow nanopillars, together with further microstructural analysis, can be found in [243]. From this work, the high degree of crystallinity of the vertically grown tungsten-based nanowires can be seen in the high-resolution STEM image of Figure 7k. The fabrication of elegant 3D superconducting tungsten-based nanohelices has also been demonstrated, achieving ultrasmall helix diameters and pitch [244] (Figure 7l). In another helium FIBID superconductivity application, this time using in-plane deposition, tungsten-based nanowires up to 390 μm in length were deposited in order to connect to niobium thin-film islands, to fabricate hybrid microwave resonators [249]. The superconducting properties of tungsten-based in-plane helium-FIBID nanowires have been further investigated in [234].

Finally, experimental investigations supported by Monte Carlo simulations are underway to enable helium FIBID of complex nanopillar-based 3D mesh objects with sizes in the submicrometer domain [245] (Figure 7m).

## Conclusion and Outlook

The impact of the helium ion microscope, since it was introduced in 2007, has been far-reaching. In addition to its unique properties as an imaging instrument, it has enabled fundamental study of a variety of physical processes and the fabrication of nanoscale structures and devices not possible by other means. As this review article has described, these applications make use of localized irradiation over a wide range of ion doses, generating point defects for various forms of defect engineering, implanting ions to form nanobubbles for basic research into the irradiation response of materials, inducing subsurface swelling for patterning by nanoscale tumefaction, re-structuring materials externally by ion-induced mass transport and internally by collisional mixing, inducing stress gradients for controlled deformation of membranes, patterning resists for advanced ion beam lithography, and at the highest doses, selectively removing material by ion milling and adding material by gas-assisted deposition to fabricate a range of nanostructures for device prototyping that is truly at the cutting edge.

Future exciting avenues to pursue include continued development of in situ platforms for additional control over the sample environment and for real-time characterization of property effects, novel applications of irradiation-induced phenomena and FIB-based nanofabrication aided by extension of the available energy range of the ions to both higher and lower values, and further development of 3D nanofabrication techniques to propel breakthroughs in the 3D nano realm.
